# A pilot study of the use of the oral and faecal microbiota for the diagnosis of ulcerative colitis and Crohn's disease in a paediatric population

**DOI:** 10.3389/fped.2023.1220976

**Published:** 2023-11-16

**Authors:** A. Monleón-Getino, G. Pujol-Muncunill, J. Méndez Viera, L. Álvarez Carnero, W. Sanseverino, A. Paytuví-Gallart, J. Martín de Carpí

**Affiliations:** ^1^ Department of Genetics, Microbiology and Statistics, Universitat de Barcelona, Barcelona, Spain; ^2^ GRBIO, Research Group in Biostatistics and Bioinformatics, Barcelona, Spain; ^3^ BIOST3, Research Group in Biostatistics, Data Science and Bioinformatics, Barcelona, Spain; ^4^ Unit for the Comprehensive Care of Paediatric Inflammatory Bowel Disease, Paediatric Gastroenterology, Hepatology and Nutrition Department, Hospital Sant Joan de Déu, Barcelona, Spain; ^5^ Sequentia Biotech SL, Barcelona, Spain

**Keywords:** metagenomic, Crohn's disease, ulcerative colitis, diagnosis, biodiversity

## Abstract

Crohn's disease (CD) and ulcerative colitis (UC) are chronic inflammatory bowel diseases (IBD) that affect the gastrointestinal tract. Changes in the microbiome and its interaction with the immune system are thought to play a key role in their development. The aim of this study was to determine whether metagenomic analysis is a feasible non-invasive diagnostic tool for IBD in paediatric patients. A pilot study of oral and faecal microbiota was proposed with 36 paediatric patients divided in three cohorts [12 with CD, 12 with UC and 12 healthy controls (HC)] with 6 months of follow-up. Finally, 30 participants were included: 13 with CD, 11 with UC and 8 HC (6 dropped out during follow-up). Despite the small size of the study population, a differential pattern of microbial biodiversity was observed between IBD patients and the control group. Twenty-one bacterial species were selected in function of their discriminant accuracy, forming three sets of potential markers of IBD. Although IBD diagnosis requires comprehensive medical evaluation, the findings of this study show that faecal metagenomics or a reduced set of bacterial markers could be useful as a non-invasive tool for an easier and earlier diagnosis.

## 1. Introduction

The human gut is the natural habitat for a large and dynamic bacterial community. Recent studies indicate that the gastrointestinal tract is colonized by about 10^13^–10^14^ microorganisms, the highest bacterial content being found in the colon (1). Inflammatory bowel disease (IBD) is a chronic inflammatory condition of the gastrointestinal tract, which is thought to arise from altered interactions between gut microbes and the intestinal immune system (2–4). In recent years, metagenomics and other omics methodologies have been used to understand IBD pathogenesis (5–8).

In the present study, oral and faecal bacterial microbiota were investigated to characterize the metagenomic diversity of the gut microbiota in paediatric IBD patients. Changes in the diversity of the microbiota were assessed and machine learning classification algorithms were used to determine possible sets of bacterial markers of the two subtypes of IBD, Crohn's disease (CD) and ulcerative colitis (UC), and assess their performance.

### 1.1. Background and status

In the last fifteen years, the global incidence of IBD has increased in both adult and paediatric populations, with approximately 20%–25% of patients with IBD diagnosed before the age of 18. In 2013, Martin *et al*. found that the incidence in children and adolescents had tripled in 13 years, increasing from 0.97 to 2.8 cases per 100,000 inhabitants under 18 years of age per year of the study period (9).

Although the true aetiology of IBD remains unknown, it is thought to be triggered by lifestyle factors in Western societies (diet, infectious agents, and environmental stimuli) in genetically predisposed individuals (10–13). The gut microbiota plays an important role in maintaining intestinal homeostasis, and major shifts in composition can lead to dysbiosis and an abnormal immune response (14–19). Studies comparing the gut of paediatric IBD patients and healthy individuals have revealed differences in both the profile and abundance of microorganisms [Lloyd-Price, Nature 2019; (3) Cell Host Microbe].

The diagnosis of IBD in paediatric populations involves clinical examination, laboratory tests, upper and lower endoscopy with histological evaluation and radiological studies to evaluate small bowel peristalsis (20, 21). Diagnosis delay in children can be more than a year, with deleterious short-, medium-, and long-term consequences. Therefore, a high level of disease awareness is essential for early detection and to establish the appropriate treatment as soon as possible (22). In this way, the impact of the disease on patient growth, nutrition, and pubertal development is minimized (23).

### 1.2. Objectives and hypothesis

The identification of specific biomarkers and new diagnostic targets in IBD are important to further our understanding of the disease and improve diagnosis. The aim of the study was to use metagenomics to characterise the biodiversity of the gut microbiota in paediatric IBD patients and assess whether microbiome data could be used as a rapid non-invasive diagnostic tool to complement the currently available invasive and non-invasive techniques (laboratory, endoscopy, histology, and radiology).

We hypothesized that the microbiome signature differs between paediatric IBD (CD and UC) patients and healthy controls in saliva and stools sampled at the initial diagnosis, but the differences are not maintained in the gut microbiome at 6 months of follow-up if the patient is in clinical remission.

## 2. Material and methods

### 2.1. Ethical statement

The study was approved by the Ethics Committee of the Hospital Sant Joan de Déu, according to Spanish biomedical law 14/2007 (reference number PIC-19-18).

Personal data were treated according to the Spanish law of personal data protection and guarantee of digital rights [Ley de Protección de Datos y Garantía de Derechos Digitales (LOPDGDD), BOE-A-2018-16673].

### 2.2. Patient recruitment and cohort design

A prospective, longitudinal observational pilot clinical trial was performed with the consecutive inclusion of paediatric IBD patients matched with HC individuals by age and sex. Initially, a total of 36 children were included: 12 CD patients, 12 UC patients and 12 HC. Demographic and clinical data were compiled from medical reports, and blood was collected from the IBD patients at different time points (0, 3 and 6 months) to be tested for inflammatory markers: C-Reactive Protein (CRP), erythrosedimentation rate (ESR), and faecal calprotectin (FC). Stool and saliva samples were collected from all participants at baseline, and from CD and UC patients also at 3 and 6 months for DNA sequencing and bioinformatic analysis.

Clinical remission was defined by a Paediatric Crohn's Disease Activity Index (PCDAI) of <12.5 for CD patients and a Paediatric Ulcerative Colitis Activity Index (PUCAI) < 10 for UC patients.

Written informed consent was obtained from parents and informed assent from patients older than 12 years before inclusion in the study or collecting the data on the Case Report Form (CRF).

#### 2.2.1. Eligibility criteria

We consecutively included patients with CD or UC from 6 to 18 years of age, who were diagnosed by the Porto Criteria and signed the informed consent to participate in the study. The control participants were children without any known digestive disease who visited the outpatient clinic for other reasons. Exclusion criteria were therapy with antibiotics or probiotics in the month before the baseline tests.

#### 2.2.2. Sample collection

Oral and faecal samples were taken in all patients. Stool collection was carried out by the parents at home or in the hospital if the patient was admitted using the microbiome collection kit (OM-200, DNA Genotek Inc., Ottawa, ON, Canada). Beforehand, the collection process and use of the kit were briefly explained to the parents. Stool collection was performed not more than 24 h prior to the oral sample collection, and all samples were kept and stored at (−20 ± 5)°C until DNA extraction. The oral samples were collected by trained healthcare professionals in the hospital using the ORAcollect for Pediatrics (OC-175, DNA Genotek Inc., Ottawa, ON, Canada) and stored at (−20 ± 5)°C until DNA extraction.

At baseline, and at 3 and 6 months, a blood test for inflammatory markers (CRP and ESR) and FC was performed in the CD and UC cohorts.

### 2.3. DNA purification and NGS sequencing

Automated DNA extraction from oral and faecal samples was carried out by Laboratorio Echevarne (Barcelona, Spain) following instructions of the kit manufacturers. A QIAsymphony DSP Virus/Pathogen Kit (QIAGEN Iberia, S.L., Barcelona, Spain) in combination with the QIAsymphony SP was used for oral samples and the QIAamp PowerFecal Pro DNA Kit (QIAGEN Iberia, S.L., Barcelona, Spain) with the QIAcube Connect for faecal samples.

DNA-seq library preparation and sequencing were carried out by IGA Technology Services (Udine, Italy). The library of each sample was constructed using a Nextera DNA Flex Library Prep Kit (Illumina, San Diego, CA, United States) following the manufacturer's instructions. Both input and final libraries were quantified by a Qubit 2.0 Fluorometer (Invitrogen, Carlsbad, CA, United States) and their quality was tested by an Agilent 2,100 Bioanalyzer High Sensitivity DNA assay (Agilent technologies, Santa Clara, CA, United States). Libraries were then prepared for sequencing and sequenced using the NovaSeq 6,000 platform in paired-end 150 mode (Illumina, San Diego, CA, United States).

The primary bioinformatic analysis included (1) base calling and demultiplexing, processing raw data for both format conversion and de-multiplexing by Bcl2Fastq version 2.20 (Illumina, San Diego, CA, United States, RRID: SCR_015058); and (2) masking of adapter sequences with Cutadapt version 1.11 (24) from raw fastq.

### 2.4. Metagenomic analysis

Starting from the raw reads, taxonomic profiling was carried out with the cloud software GAIA (v 2.02) (25) to obtain operational taxonomic unit (OTU) tables at different taxonomic levels. Their corresponding Shannon alpha-diversity and Bray-Curtis beta-diversity values were computed with the R package phyloseq (26). The functional profiling was performed by first aligning the reads with diamond (27) against the UniRef90 (release 2019_06) (28) protein database. Multi-mapping reads to different proteins were assigned to a unique protein with FAMLI (29). Finally, different functional information from the proteins such as gene ontology terms or pathways were downloaded using the UniProt API. These last analyses will not be presented in this work, but in the future when properly processed.

### 2.5. Statistical analysis

For this study, general statistical methods were used to analyse the metagenomic results obtained in the form of OTU frequency tables, such as descriptive methods for frequencies, and a statistical test of binomial proportions to compare the frequencies between experimental groups. Other multivariate analyses were also used to evaluate the different hypotheses and objectives of the study, namely, principal component analysis (PCA), estimation of alpha and beta biodiversity measures, and representation of the relationships between types of bacteria for each experimental group in the form of a network.

Statistical analyses were performed using different R functions and libraries (30). The BDbiost3 library for R (31, 32) was used to assess the coverage of the sequenced reads [function PILI3()] and for discriminant and exploratory data analysis. All statistical procedures that have been used in this investigation such as: (a) Biodiversity and Venn diagrams analysis using functions Analysis.Biodiver.Metagen(), Betabiodiversity() and coincidence.analysis(); (b) hypothesis test for the OTU proportions comparation between experimental groups using function dif. propOTU.between.groups (); (c) Discriminant and exploratory data analysis using function ANNA.DISCRIMINANT.MaLearning.Predict (); (d) Spectral clustering network analysis and identification of communities (consortiums) between bacterias using functions Espectral.CN() and Miriam.Network() are conveniently described in more detail in the Supplementary Material.

## 3. Results

### 3.1. Cohort description

Finally, 24 IBD patients were included in the study (13CD, 11UC) and 8 HC. Six patients dropped out before completing 6 months of follow-up for reasons associated with the COVID-19 pandemic: 2 of them at 3 months and the other 4 at 6 months. Table 1 presents the principal descriptive characteristics for the experimental (CD, UC) and control groups (HC). As can be seen, the sociodemographic characteristics (sex, ethnicity, and age) between groups are quite similar.

**Table 1:** Demographic characteristics of experimental and control groups.

Group	% Sex (male)	% Ethnicity (Caucasian, Arabian, Hispanic)	Age (Median, min, max) years
HC (controls) (*n* = 8)	44.4%	100%, 0%, 0%	12 (10–16)
UC (*n* = 11)	54.5%	72.7%, 9.1%, 18.2%	13 (9–15)
CD (*n* = 13)	61.5%	92.3%, 7.8%, 0%	11 (8–15)

Table 2 presents the clinical results evolution (diagnosis, 3 m, 6 m) for Pediatric Crohn's Disease Activity Index (PCDAI), Pediatric Ulcerative Colitis Activity Index (PUCAI), Fecal calprotectin (FC), erythrocytic sedimentation rate (ESR) and C-reactive protein (CRP) during the clinical progression of children. We stratified patients into 3 subgroups based on the FC results: FC1 < 250 mg/kg (possible remission), FC2 from 250 mg/kg to 500 mg/kg (grey zone), and FC3 > 500 mg/kg (possible inflammatory activity).

**Table 2:** Summary of the clinical evolution of different common markers in crohn's disease (CD) and ulcerative colitis (UC) at diagnosis, 3 months and 6 months.

Clinical group	Timeline	PCDAI/PUCAI mean (IQR)	FC (mg/kg)	FC stratified (%)			EIM (ESR and/or CRP) (%)
				FC1	FC2	FC3	
CD	Diagnosis (*n* = 13)	25 (10–47.5)	2,166 (159–6,000)	0% (*n* = 0)	0% (*n* = 0)	100% (*n* = 13)	83%
	3 m (*n* = 12)	0 (0–0)	281 (50–2,880)	50% (*n* = 6)	17% (*n* = 2)	33% (*n* = 4)	0%
	6 m (*n* = 11)	0 (0–0)	93 (6–1,973)	64% (*n* = 7)	0% (*n* = 0)	36% (*n* = 4)	0%
UC	Diagnosis (*n* = 11)	30 (10–80)	3,381 (526–6,000)	0% (*n* = 0)	0% (*n* = 0)	100% (*n* = 11)	18%
	3 m (*n* = 10)	0.5 (0–5)	33 (7–1,042)	90% (*n* = 9)	0% (*n* = 0)	10% (*n* = 1)	0%
	6 m (*n* = 7)	0.5 (0–5)	9 (0–380)	90% (*n* = 6)	0%	10% (*n* = 1)	3.3%

IQR, Interquartile range; PCDAI, Pediatric Crohn's Disease Activity Index; PUCAI, Pediatric Ulcerative Colitis Activity Index; FC, Fecal calprotectin; ESR, erythrocytic sedimentation rate; CRP, C-reactive protein; EIM, elevation of inflammatory markers.

It is worth noting that at diagnosis, 83% of CD patients presented elevated inflammatory markers (ESR and/or CRP) and 100% present possible inflammatory activity (FC3). At 3 and 6 months, all CD patients were in clinical remission with blood inflammatory markers within normal limits. At 3 and 6 months, respectively, only 33% and 26% of patients were in FC3 (see Table 2). Regarding the treatment used in the CD cohort, 7/13 patients received exclusive enteral nutrition for 8 weeks with azathioprine to induce remission and 6 patients needed biological therapy at diagnosis (5 Adalimumab, 1 Infliximab), 3 of them in combotherapy with azathioprine. At 3 and 6 months, no changes in treatment were required.

At diagnosis the 18% UC patients had elevated inflammatory markers (ESR and/or CRP) and 100% present possible inflammatory activity (FC3). At 3 months, all patients were in clinical remission, which was maintained at 6 months in all patients except one (PUCAI = 30). No blood inflammatory markers were elevated at 3 months, and only one patient had elevated CRP at 6 months. At 3 and 6 months, respectively, only 10% of patients were in FC3 (see Table 2). Regarding the medication used in the UC cohort during induction, 8 patients received mesalazine as first line therapy and 3 patients required steroids and azathioprine. At 3 months, 4 patients required initiation of biological therapy with anti-TNF (infliximab) and 3 added azathioprine due to inflammatory activity, maintaining the same treatment until the end of the follow-up.

### 3.2. General description of the sequencing results

A total of 497,074,958 reads were generated from 143 samples (256,071,356 for faecal samples and 241,003,602 for oral samples), with an average of 3,476,049 reads per sample. A total of 415,140,921, 377,589,515 and 332,954,508 reads were selected for bacterial family, genus and species, respectively, after quality and microbiology processing. Read representativeness with respect to OTU biodiversity was very high, ranging from 99% to 100% (Supplementary Figure 1) for the main experimental groups tested. The OTU coverage obtained was close to 100%, indicating that the obtained reads reflected the expected diversity in these samples. The total number of bioinformatically identified species was 240, once the number of unknown OTUs was reduced to 236, corresponding to 46 families, 74 genera and 138 species (Supplementary Tables 1–3).

For the analysis and statistical comparisons, the patients, samples, and time of sample collection are referred to using combinations of the following abbreviations. Besides the HC, UC, and CD cohorts, OR and FE refer to oral and faecal samples, respectively, and the times of sample collection are baseline (DE), and 3 (3M) and 6 months (6M) of treatment.

### 3.3. Biodiversity, bacterial distribution

Richness (S, JACKNIFE2, CHAO) and abundance (OTUs) were calculated, and the main biodiversity indices (Shannon's index [H], Simpson's index [simp], inverse of Simpson's index [invsimp], and alpha index [alpha]) and evenness [J Pielou's index]) were obtained. The values for each sample and the statistical comparisons of biodiversity are shown in Supplementary Table 4. Bacterial biodiversity in the HC cohort was higher compared to the IBD patients at family, genus, and species levels, but without statistical significance.

A PCA dimension reduction analysis was used to facilitate clinical and microbial characterization using the majority of variables collected with the experimental groups and these results are summarized in Figures 2, 3.

### 3.4. Characterization of bacterial populations through inferential statistical analysis

Different inferential statistical techniques were used to explore if bacterial groups show statistically significant differences when cohorts and different stages of clinical treatment are compared. The OTU counts were transformed into percentages and a comparison test of binomial proportions, corrected for the multiplicity of tests, was applied. Each individual sample was compared (oral, faecal, CD and UC). Table 3 summarizes the more than 6,000 statistical tests (binomial proportion tests) performed to detect the differential proportions of bacterial species, genera, and families between experimental groups. Additionally, the control samples are compared with those taken at DE, 3M and 6M. Supplementary Tables 1–3 show the results of the binomial tests applied to the percentages of bacterial species, genera, and families, respectively. The discussion of this results can be found in the Supplementary Material.

**Table 3:** Differential results of the statistical tests between groups using the binomial proportions test.

	Taxon	All *p*-values computed ≤4 Groups (CD, UC, OR, FE) ≤6 Pairs (HC, DE, 3M, 6M)	Significant tests (*p* < 0.05) Percent respect total tests	Significant difference of proportions ≥2%, ≥5%, ≥10% between groups Percent respect total tests
All	Species	24 (g × p) × 138 sp. = 3,312 tests	3,115 (94.1%)	262 (7.9%), 50 (1.5%), 30 (0.9%)
	Genus	24 (g × p) × 74 gen. = 1,776 tests	1,642 (92.4%)	164 (9.2%), 40 (2.3%), 40 (2.3%)
	Family	24 (g × p) × 46 fam. = 1,104 tests	1,025 (92.8%)	172 (15.6%), 35 (3.2%), 38 (3.4%)
Faecal	Species	12 (g × p) × 138 sp. = 1,656 tests	1,512 (91.3%)	154 (58.7%), 33 (66%), 28 (93.4%)
	Genus	12 (g × p) × 74 gen. = 888 tests	791 (89.1%)	81 (50.1%), 23 (57.5%), 25 (62.5%)
	Family	12 (g × p) × 46 fam. = 552 tests	496 (89.9%)	85 (49.6%), 21 (60.2%), 22 (58.2%)
Oral	Species	12 (g × p) × 138 sp. = 1,656 tests	1,603 (96.8%)	131 (49.9%), 25 (34%), 16 (6.6%)
	Genus	12 (g × p) × 74 gen. = 888 tests	851 (95.8%)	83 (50.8%), 20 (42.5%), 21 (37.5%)
	Family	12 (g × p) × 46 fam. = 552 tests	529 (95.8%)	88 (51.4%), 19 (40.2%), 21 (41.1%)
UC-FE	Species	6 (g × p) × 138 sp. = 828 tests	762 (92.02%)	80 (40.4%), 18 (36%), 15 (50.1%)
	Genus	6 (g × p) × 74 gen. = 444 tests	398 (89.6%)	43 (26.30%), 13 (32.50%), 14 (35.00%)
	Family	6 (g × p) × 46 fam. = 276 tests	247 (89.5%)	43 (25.10%), 12 (34.40%), 13 (34.40%)
CD-FE	Species	6 (g × p) × 138 sp. = 828 tests	750 (90.6%)	74 (28.30%), 15 (30.00%), 13 (43.30%)
	Genus	6 (g × p) × 74 gen. = 444 tests	393 (88.5%)	39 (23.80%), 10 (25.00%), 11 (27.50%)
	Family	6 (g × p) × 46 fam. = 276 tests	249 (90.2%)	42 (24.50%), 9 (25.80%), 9 (23.80%)
UC-OR	Species	6 (g × p) × 138 sp. = 828 tests	791 (95.5%)	57 (21.80%), 10 (20.00%), 1 (3.30%)
	Genus	6 (g × p) × 74 gen. = 444 tests	418 (94.1%)	42 (25.60%), 10 (25,00%), 8 (20.00%)
	Family	6 (g × p) × 46 fam. = 276 tests	259 (93.8%)	42 (24.50%), 7 (20.10%), 8 (21.10%)
CD-OR	Species	6 (g × p) × 138 sp. = 828 tests	812 (98.1%)	51 (19.50%), 7 (14.00%), 1 (3.30%)
	Genus	6 (g × p) × 74 gen. = 444 tests	433 (97.5%)	40 (24.50%), 7 (17.50%), 7 (17.50%)
	Family	6 (g × p) × 46 fam. = 276 tests	270 (97.8%)	45 (26.30%), 7 (20.10%), 8 (21.00%)

### 3.5. Discriminant analysis of species that differentiate between baseline CD and control faecal samples

To determine which bacterial species could potentially serve as markers to detect CD in its early stages, a stepwise linear discriminant analytical method using the package *caret* for R (33) was used. The discriminant analysis was carried out between faecal samples from the control cohort and those collected at baseline from CD patients. The number of discriminating bacteria was limited to a maximum of 10 species. All the results are presented in Table 4.

**Table 4:** Set of discriminating bacteria between control and faecal-CD groups.

	Algorithm	Discriminant accuracy (CI 95%) Training (80%)/Test (20%)		Sensitivity and specificity Training (80%)/Test (20%)	
Set 1	SVM	1.00 (0.85, 1.00)	0.59 (0.36, 0.79)	1, 1	–, 0.59
	KER	0.95 (0.77, 0.99)	0.90 (0.71, 0.99)	0.89, 1.00	0.78, 1.00
	LDA	1.00 (0.85, 1.00)	1.00 (0.84, 1.00)	1.00, 1.00	1.00, 1.00
Set 2	SVM	0.59 (0.36, 0.79)	0.91 (0.71, 0.99)	–, 0.59	1.00, 0.87
	KER	0.91 (0.71, 0.99)	0.86 (0.65, 0.97)	0.78, 1.00	0.78, 0.92
	LDA	0.81 (0.60, 0.95)	0.81 (0.60, 0.95)	0.78, 0.85	0.78, 0.85
Set 3	SVM	0.91 (0.71, 0.99)	0.91 (0.71, 0.99)	1.00, 0.87	1.00, 0.87
	KER	0.86 (0.65, 0.97)	0.95 (0.77, 0.99)	0.67, 1.00	0.89, 1.00
	LDA	0.95 (0.77, 0.99)	0.95 (0.77, 0.99)	0.89, 1.00	0.89, 1.00

The accuracy values of the discriminant method and its specificity and sensitivity are presented.

SVM, support vector machine; KER, kernel discriminant; LDA, linear discriminant analysis % indicate that original set was converted in percentages.

mg/kg→possible activity (3)).

## 4. Discussion

Although several studies on IBD have applied a metagenomics approach, few have focused on a paediatric population. In the present work, differential microbiota biodiversity was characterized in children affected by CD and UC. It was possible to differentiate the control cohort from the IBD patients before treatment based on beta biodiversity. Additionally, changes in diversity were observed during the clinical evolution of the patients. Alpha biodiversity was higher in the control cohort and in oral vs. faecal samples.

### 4.1. Richness differential microbiota pattern

In CD patients, the first simple statistical analysis revealed a differential microbiota pattern (Supplementary Figure 1) in both oral and faecal samples at all collection times in comparison with HC individuals. In faecal samples, 3 differential taxa were found at baseline and 3 months, and oral samples contained 3 other differential taxa. In UC patients, 6 differential taxa (>3% diff. HC-UC, *p*-value = 0) were observed in faecal and 7 in oral samples at baseline compared to the HC samples. Globally, a higher microbial biodiversity was observed in HC individuals compared to CD patients at disease onset, but the difference was not statistically significant.

The distribution of bacterial species per sample type and cohort was analysed to observe any differences or patterns. Supplementary Figure 1 provides a graphical summary (circular barplots) of the distribution of the percentages of each microbial taxon (family, genus, species) with a minimal frequency of 1% for genera and families and 2% for species. In CD patients, a differential microbiota pattern is apparent between the two sample types (oral and faecal) at each sampling point (DE, 3M and 6M). The microbiological evolution in IBD samples, observed by comparing the taxa at the three sampling points, differed from the HC pattern. Notably, *Streptococcus sanguinis* was observed in faecal samples of CD and UC patients at DE and 3M but not in HC individuals. Changes in species richness in the different sample groups (CD, UC, OR, FE) during the clinical evolution was analysed using Venn diagrams (data not shown), and the number of species at DE, 3M or 6M did not differ significantly from HC samples.

### 4.2. Clinical and biodiversity importance

**Figure 1:**
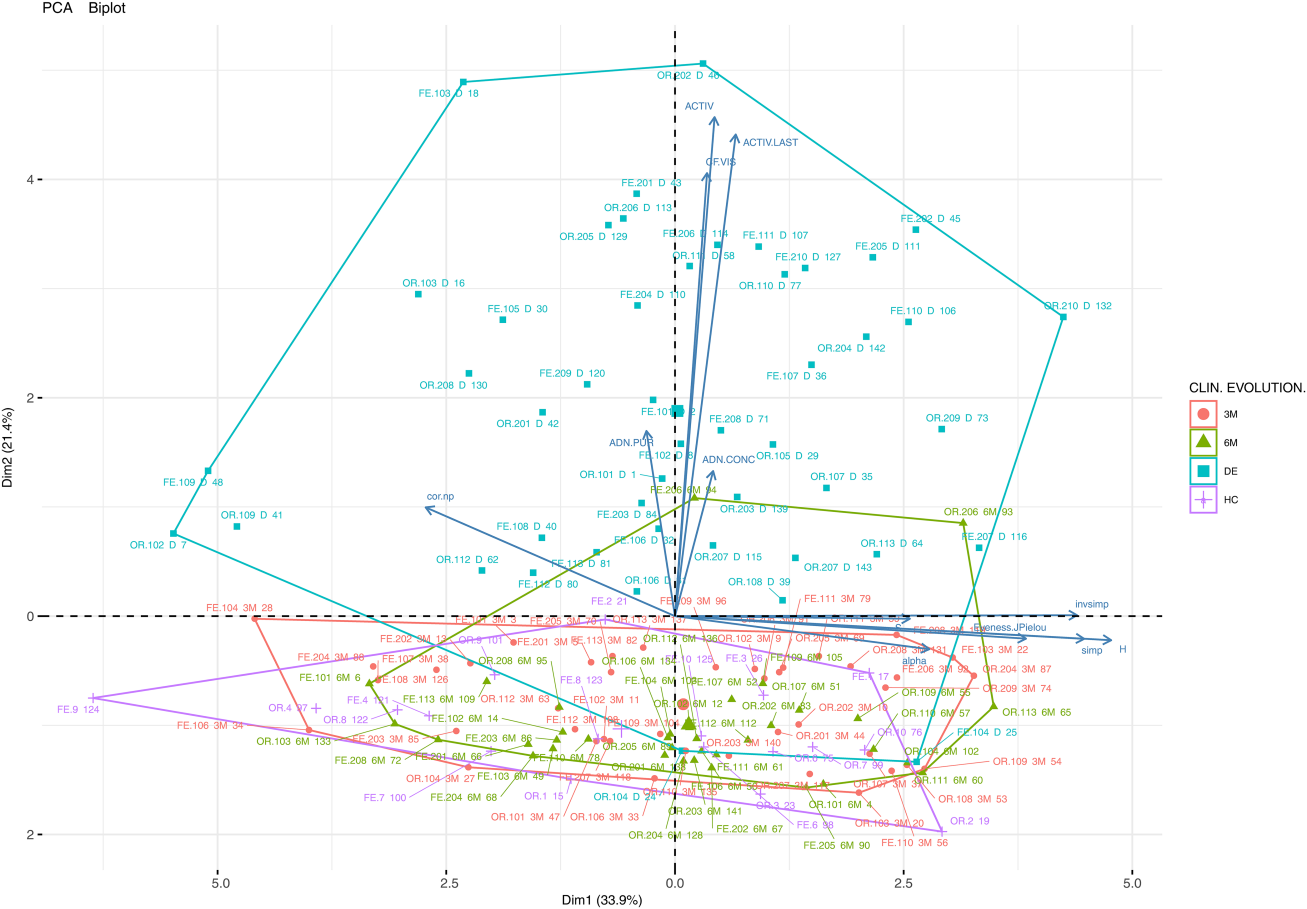
Principal component analysis (PCA) biplot (variables and cases) of the clinical, biodiversity (richness, abundance, biodiversity indices) and other variables (DNA concentration, DNA purity) of all the samples and experimental groups of the study (CD, Crohn's disease; UC, ulcerative colitis; OR, oral samples; FE, faecal samples; HC, healthy control; DE, sampling points at disease onset/baseline; 3M, 3 months; 6M, 6 months). A point has been plotted for each of the 143 samples using sample type (FE, OR) and sample number (e.g., 102-DE, patient 102 at disease onset).

Figure 1 shows a two-dimensional PCA analysis of the data for the main studied variables: DNA concentration, DNA purity, alpha biodiversity (abundance and richness, biodiversity indices), clinical activity and FC in individual samples (36 experimental groups: UC, CD, OR, FE, DB, HC) and the Bray-Curtis distance between the control (HC) and other samples (cor.np, e.g., correlation between HC and patient oral samples). The importance of each variable for the interpretation of the components (biplot) is also represented. The samples clustering on the left of the horizontal axis constitute the first component (Dim1), which explains 33.9% of the total variability and can be interpreted as having a greater alpha biodiversity (H, inverse Simpson). The second component (Dim2), which explains 21.4% of the total variability, can be associated with patient clinical activity and FC. Thus, the patients in the upper quadrants are those with greater clinical activity and a higher FC value.

The importance of each variable for the interpretation of the components (Dim1 and Dim2) is also represented (by arrows). The assessed samples are clustered according to their clinical evolution. Finally, it can be observed that the samples are very heterogeneous in terms of alpha biodiversity (Dim1) and there is no clear pattern regarding alpha biodiversity groupings.

The individual samples displayed in the graph are identified according to their origin (oral or faecal), the patient type (CR or UC), and time of collection (DE, 3M or 6M). The analysis reveals that patient samples collected at baseline (mainly placed in the upper quadrant) are more dispersed in terms of clinical and microbial characterization than the samples taken after treatment or from the control cohort (lower quadrant). From a clinical point of view, UC and CD patients experienced a progressive reduction in FC levels and clinical activity. Although the evolution of the microbiological community in terms of biodiversity is not clear, the samples in the right quadrant show a higher biodiversity than those in the left quadrant.

**Figure 2:**
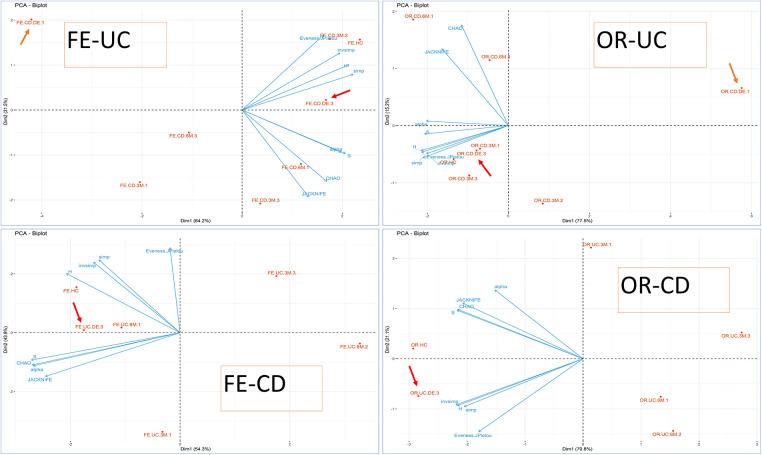
PCA biplot (biodiversity variables and cases) using PCA for groups. OR-CD and OR-UC refer to oral samples collected from CD and UC patients, respectively, whereas FE-CD and FE-UC refer to faecal samples collected from CD and UC patients, respectively, according also to biological activity (FC). The clinical activity is indicated for each point-group as a number at the end of the label. FC levels were categorized according to the range: (1) possible remission FC < 250 mg/kg, (2) grey area FC between 250 mg/kg to 500 mg/kg, and (3) possible clinical activity FC > 500 mg/kg). Arrows indicate the position of the DE, sampling points at disease onset/baseline.

To clarify this point, the previous exploratory PCA was completed by generating a 4 PCA biplot based only on biodiversity variables (richness, abundance, biodiversity indices), where samples were grouped according to their origin, type of IBD and biological activity measured by FC (FC1, FC2 or FC3). As depicted in Figure 2, in CD patients the baseline samples are far more spaced apart in terms of biodiversity than those collected during the evolution of the disease or the HC samples. Thus, the biodiversity of both oral and faecal samples at 6M is closer to HC than to DE samples. This observation does not hold for UC patients, whose samples are not/less clearly separated from those of HC. No clear pattern emerges regarding the biological activity measured by FC with respect to patient evolution, perhaps due to the low number of samples and because few patients had inflammatory activity at the end of the study (6 months).

In addition, the microbiota profile was more diverse in healthy individuals than in patients at baseline as well as at 3 or 6 months, although the difference had decreased at the end of the follow-up. This seems to suggest that although the microbiota evolves continually during the treatment, it is only once disease remission is achieved that it becomes more complex and diverse.

### 4.3. Beta biodiversity between experimental groups and clinical association

Generally, levels of beta biodiversity in faecal samples are too erratic to be useful for clinical monitoring. However, certain associations observed were consistent with the hypotheses raised in the study. Thus, the microbiota of the control group clearly had a higher similarity to that of sick children after 6 months of treatment than at baseline.

**Figure 3:**
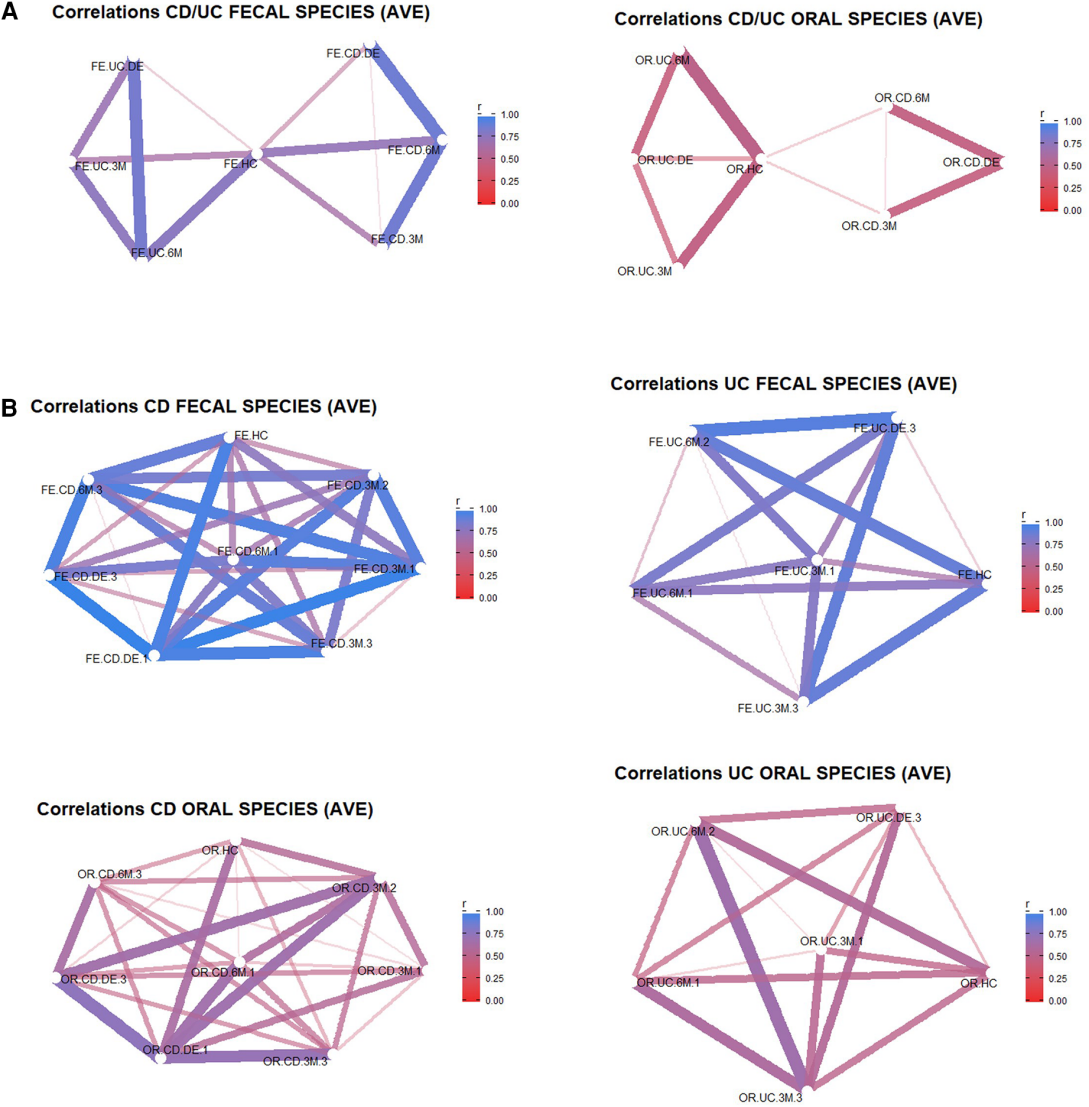
Bray-Curtis similarity networks. In (**A**) the networks are between the different experimental groups during the clinical evolution of the patients with respect to the controls for faecal samples (left) and oral samples (right). In (**B**) the networks are between aggregated samples according to the different groups (CD/UC, HC, DE, 3M, 6M) and categories according to IBD type (UC or CD) and FC activity (FC <250 mg/kg→possible remission (1), FC 250–500 mg/kg→grey area (2), FC > 500.

A network analysis (Figure 3) was carried out from the Bray-Curtis distances between pairs of samples, which were grouped according to the time of collection (HC, DE, 3M and 6M) and FC categorization. The objective of these analyses was to assess the distance between the control samples and those collected at disease onset, and thus to determine if the level of similarity to the control is higher for DE or 6M samples. The importance of each variable for the interpretation of the components (represented as arrows) is plotted in the graph. It is noteworthy that the DE samples (both oral and faecal) of IBD patients appear closer to the control samples than the 6M samples only in CD patients.

Figure 3A shows the Bray-Curtis similarity in terms of bacterial abundance. The faecal samples collected from the control cohort have less similarity with those of the IBD patients (both UC and CD) at disease onset than after 6 months of treatment. However, this relationship is not apparent for oral samples, with similarity indices close to 0. In Figure 3B, the experimental groups are categorized according to the three classes of the analytical variable FC. Again, the similarity (beta biodiversity) between the control cohort and IBD patients is lower at disease onset than after 6 months of treatment. Samples belonging to IBD patients with the highest FC levels (250 mg/kg to 500 mg/kg or values > 500 mg/kg) show less similarity with regard to the control than those with the lowest FC value. This indicates that the biodiversity of IBD patients with low FC levels more closely resembles the biodiversity of the control group. When the same analysis (data not shown) was applied to aggregated UC and CD samples at disease onset, the similarity in bacterial abundance between UC and CD faecal and oral samples was reduced.

In summary, when all the groups and categories are compared with the control cohort, the main difference in beta biodiversity is apparent at the initial diagnosis of IBD. After clinical treatment, the differences decreased, especially in patients with low FC levels, although this reduction may be difficult to infer for UC patients as the reduced number of samples resulted in an unbalanced group. In addition, a higher biodiversity was observed in faecal than in oral samples.

When microbial populations were characterized by inferential statistics and discriminant analysis (see Table 3 and Supplementary Material for interpretation), most bacteria were associated with pathogenicity, inflammation, or biofilm formation, although a small number were related with a reduction of inflammation. These results suggest the bacteria may be involved in the dysbiosis of IBD patients.

### 4.4. A tool for an easier and earlier diagnosis based on discriminant analysis

Three sets of discriminant bacteria were selected that includes expert criteria, after many scenarios in the previous phases of the discriminant analysis (feature selection), that were selected according to their accuracy, sensitivity, and specificity to detect CD in its early stages. Set 1 is composed of *Neisseria.subflava*, *Eubacterium rectale*, *Capnocytophaga sputigena*, *Ruminococcus torques*, *Abiotrophia defectiva*, *Cutibacterium acnes*, *Bifidobacterium breve*, *Streptococcus thermophilus*, *Neisseria elongate* and *Actinomyces naeslundii*. Set 2 contains *Neisseria subflava*, *Fusobacterium nucleatum*, *Eubacterium siraeum*, *Phyllobacterium zundukense*, *Abiotrophia defectiva*, *Aggregatibacter aphrophilus*, *Acidaminococcus intestini*, *Bifidobacterium pseudocatenulatum*, *Fusobacterium periodonticum* and *Rothia mucilaginosa*.

Finally, set 3 is composed of *Neisseria subflava*, *Ornithobacterium rhinotracheale*, *Enterococcus faecium*, *Bacteroides caecimuris*, *Ruminococcus torques*, *Streptococcus thermophilus*, *Streptococcus* sp. oral taxon 064, *Abiotrophia defectiva*, *Actinomyces naeslundii* and *Actinomyces* sp. oral taxon 414.

Concerning *Ornithobacterium rhinotracheale*, a misclassification may have occurred, and it could be *Candidatus Ornithobacterium hominis,* which shares ca. 93% of nucleotide identity and frequently colonizes the infant nasopharynx (34).

All these discriminant sets were tested using three different discriminant methods (SVM, support vector machine; KER, kernel discriminant; LDA, linear discriminant analysis) to test confidence interval accuracy, sensitivity and specificity. The original discriminant set was converted in OTU percentages or kept as OTU counts.

Table 4 present the discriminating results [Discriminant accuracy (CI 95%) for training and test sets, and the sensitivity and specificity] for each bacteria sets, being the confidence interval for an accuracy of 0.85–1 in the best of the scenarios (using LDA method and test set), which indicates that it is a good discriminant method and a candidate to be used as a diagnostic method to detect CD in its early stages.

Interestingly, some of these bacteria considered are related to inflammatory processes, biofilm formation or pathogenesis (35, 36–44).

## 5. Conclusions

Although the microbiota of children was observed to evolve and the complexity of the microbiota (consortia and relationships between microorganisms) appeared lower at disease onset than at the end of follow-up, a higher number of samples and a longer study period is necessary to verify this.

Once validated, the sets of bacteria identified in the discriminant analysis could be used to build a diagnostic test that allows rapid identification of IBD and monitoring of its evolution.

## Data Availability

The datasets presented in this study can be found in online repositories. The names of the repository/repositories and accession number(s) can be found in the article/Supplementary Material.
