# Supplementary material for: A pilot study of the use of the oral and faecal microbiota for the diagnosis of ulcerative colitis and Crohn's disease in a paediatric population
Source: Front Pediatr. 2023 Nov 16;11:1220976. doi: 10.3389/fped.2023.1220976 (PMC10687547; doi:10.3389/fped.2023.1220976)
Supplement: Supplementary file 1 [file Datasheet1.pdf]

Supplementary Material

A pilot study of the use of the oral and faecal microbiota for the diagnosis of ulcerative colitis and Crohn's disease in a paediatric population

A. Monleón-Getino[1,2,3,*], G. Pujol-Muncunill[4], J. Méndez Viera[1,3], L. Álvarez Carnero[2], W. Sanseverino[3], A. Paytuví-Gallart[3], J. Martín de Carpí[2]

[1]Department of Genetics, Microbiology and Statistics, Universitat de Barcelona, Barcelona, Spain, [2]GRBIO, Research Group in Biostatistics and Bioinformatics, Barcelona, Spain, [3]BIOST3, Research Group in Biostatistics, Data Science and Bioinformatics, Barcelona, Spain, [4]Unit for the Comprehensive Care of Paediatric Inflammatory Bowel Disease, Paediatric Gastroenterology, Hepatology and Nutrition Department, Hospital Sant Joan de Déu, Barcelona, Spain, [5]Sequentia Biotech SL, Barcelona, Spain.

*** Correspondence:**
Monleón-Getino A
amonleong@ub.edu

1. Supplementary Data

This section describes additional data of interest about the study carried out.

Data Availability Statement

The raw data from which this analysis was carried out can be found in the repository, at https://doi.org/10.34810/data593

1.1 Metagenomics of the human microbiota

Changes in gene transcription are fundamental for the evolution of species. On the other hand, the conservation of regulatory mechanisms of gene expression has shed light on the evolution of human biology and disease when analysed by genetic models and comparative genomics.

The composition of microbial communities in the human body has attracted growing interest, especially in terms of their genetic characterization. Advances in DNA sequencing and the use of metagenomics and metatranscriptomics have made it possible to study complex microbial ecosystems without needing to isolate their members (1-4).

In the field of IBD, CD and UC patients are known to have a gut microbiome that differs significantly from that of healthy individuals. Various studies have shown that CD is related to a mismatch between the host and intestinal bacterial flora, whose structure and function have been characterized by the application of metagenomics and transcriptomics (5-6). Such an ecosystems biology approach has highlighted the link between the gut microbiota and functional alterations in the pathophysiology of CD and IBD in general.

The composition of the gut microbiome changes with age; in infants, most studies show that it resembles that of the adult at around the age of 3 (7). The infant gut is colonised by environmental microbes at birth, most of them originating from the mother's microbiome (vaginal, faecal, or skin) (8), the most common phyla being Firmicutes, Proteobacteria, Bacteroidetes and Actinobacteria. The most common in adults are Bacteroidetes and Firmicutes, which are usually the most abundant phyla in infants by the age of one. Compared with healthy children, paediatric IBD patients are known to have lower levels of several beneficial bacteria, such as the *Bifidobacterium* genus, Firmicutes phylum and those from the genera *Eubacterium*, *Ruminococcacea* and *Clostridium*, and higher levels of detrimental bacteria such as *Escherichia coli*, *Veillonellacea*, *Fusobacterium* and *Haemophilus parainfluenzae* (9). Investigations of the oral microbiome also revealed that up to 40% of paediatric CD patients had oral involvement, diversity in tongue samples being lower compared with healthy subjects (10).

1.2. Supplementary information about statistical methods

1.2.1 Biodiversity analysis

In biodiversity measurement, a quantitative estimate of biological variability is obtained to compare biological entities composed of diverse components. It is possible to distinguish between alpha diversity (diversity within a particular area, community, or ecosystem), usually measured by counting the number of taxa (OTUs) within the ecosystem (families, genera, and species), and beta diversity (difference in species diversity between ecosystems), which involves comparing the number of taxa unique to each ecosystem.

In the case of alpha biodiversity, three dimensions are distinguished: abundance, richness, and diversity. Local abundance is the relative representation of a species in a particular

ecosystem, usually measured as the number of individuals found per sample. Taxon richness is a measure of the total number of the considered taxa in a community. Diversity usually refers to both species number and equitability (or 'evenness') (11).

In the present study, alpha diversity was analysed using the function Analysis.Biodiver.Metagen() of the library BDSbiost3 (12). For alpha diversity analysis, the richness (S, JACKNIFE2, CHAO), richness indexes (Shannon index [H], Simpson index [simp], inverse of simpson [invsimp], alpha index [alpha]) and evenness (J Pielou's index) were calculated. The statistical test to compare biodiversity between groups was performed using the mcpHill function from the library simboot (13).

The beta diversity analysis was carried out using the function *Betabiodiversity*() from the library BDSbiost3 (12).and represented as a network using the function ggraph() of the library ggplot2. Finally, the function *coincidence.analysis*() of the library BDSbiost3 (12).was used to compute coincidences with Venn diagrams.

1.2.2. OTU proportions and the origin of the bacterial communities

A binomial test of proportions with adjustment of the *p*-value (*P*) ("FDR" method) for multiple hypothesis testing to avoid Type I error problems was used to assess differential proportions of OTUs among the different groups. This test was performed using the function *dif. propOTU.between.groups()* of the library BDSbiost3, which is based on Fisher's exact statistical test (12).

1.2.3. Discriminant and exploratory data analysis

Statistical analyses were performed using different R functions and libraries (14). The BDbiost3 library for R (12). was used to assess the coverage of the sequenced reads and for discriminant and exploratory data analysis.

The coverage of the sequenced reads was analysed to assess the representativeness of the obtained OTUs, as previously described (15). For this purpose, the *PILI3()* function of the BDbiost3 library function was used, which allowed the computation of the rarefaction curve between the number of reads and OTU abundance. This function was projected to an infinite rarefaction curve to verify its saturation or if it still had any margin to saturate. For the exploratory analysis, contingency tables (OTU abundance tables) were obtained separately for each sample group. These data followed a multinomial distribution (16).

and allowed us to apply an exploratory dimension reduction technique using non-metric multidimensional scaling (nMDS). Discriminant analysis was computed using the 20 most abundant OTUs and made possible by the function *ANNA.DISCRIMINANT.MaLearning.Predict ()* of the BDbiost3 library, which allowed the evaluation of 5 different discriminant methods: linear discriminant analysis (LDA), support vector machine (SVM), xboosting (Xboost), kernel discrimination (kernel) and artificial neural nets (ANN). The results also offered final classification accuracy and a confusion matrix of the different discrimination methods performed.

The relationship between the abundance (OTUs) of the species in each sample and their control (e.g., FE-DE and FE-HC groups) was mainly analysed using Bray-Curtis distance, although the non-parametric Spearman correlation (17) was also applied to assess the significance of the changes in biodiversity in relation to the different environmental variables; this statistic is referred to as np.cor in the text. As all the variables under consideration were quantitative and continuous (i.e., biodiversity, clinical activity, clinical activity at the end of the visit, DNA concentration and purity, np.cor), the related data were summarized by means of PCA. This analysis can significantly reduce the number of variables while still retaining much of the information in the original data set and graphically represent various hypotheses simultaneously. To represent the microbiological data, biodiversity, DNA concentration, np.cor, as well as some of the clinical variables of the children for all the experimental groups, PCA was carried out using the prcomp function of the library *factoextra*.

To study whether consortia or networks were formed among microorganisms of each experimental group, and as part of the interpretation of the ecology of these microorganisms, the networks formed among bacterial species, genera and families were calculated. For this, several procedures within the BDsbiost3 library were used. *Espectral.CN()* performs a spectral clustering network analysis for frequencies (taxon, omics data, counts) through sparse correlations or cosine similarity between columns (sparse matrices) with geometrical analysis and identification of communities (consortiums). *Miriam.Network()* performs a network analysis for frequencies (taxon, omics data, counts) and Gaussian graphs with the possibility of obtaining a complexity index. *Espectral.CN()* provides different procedures to separate the connections (nodes and vertices) formed between microorganisms from the correlation established between species. Each possible separation represents a consortium, and thus the main methods

used are walk trap and Girvan Newman (18), algorithms widely applied in social analysis to detect associations and user communities. We also obtained the main statistics of the resulting networks as follows: Number of connections within the network (nc), Network diameter / effective size of the network (nd), Number of formed groups (g), Connection quality (cq). The latter is a pseudo-qualitative appreciation of how bacteria are connected.

On the other hand, the calculation of a new complexity index is proposed based on the number of nodes and connections in the networks formed between the different microorganism species, using the ggraf procedure and only the 30 most abundant species (due to calculation problems of the procedure). This procedure is implemented in the function *Miriam.Network().*

1.3. Supplementary information about results

1.3.1. Characterization of bacterial populations through inferential statistical analysis

Binomial tests were used to interpret the clinical evolution of the patterns and to build a predictor that would allow these changes to be detected. A graphical summary of the results for bacterial species is provided in Supplementary Figure 2. The graph shows the main differences between bacterial species (statistically significant in terms of relative frequency) when comparing the sample groups based on cohorts and collection time. The number of bacterial species for each group is specified, including those whose frequency differs with a minimum of 2 %, 5% or 10% between groups (e.g., 3-2-1 indicates 3, 2 and 1 species with a difference > 2% > 5% and > 10 %, respectively). The graph lists the bacterial species with more than 10% of difference in frequency between groups: negative differences are in green and positive in red.

Notably, the main variations in bacterial frequency were found in faecal samples. In CD patients, a relative increase of *Streptococcus sanguinis*, *Streptococcus mitis* and *Bacteroides fragilis* was observed compared with the control group. Both streptococci species have been associated with colonization and pathogenesis, the former mainly with oral biofilm formation and infective endocarditis (19-21). The enterotoxigenicity of *Bacteroides fragilis* has been associated with the occurrence of IBD (22-24). *Phoenicola vulgatus* (*Bacteroides ovatus*) is reported to have a protective role against dextran sulfate

sodium- and lipopolysaccharide-induced colitis (25-26), although some studies indicate that both *Bacteroides vulgatus* and *Bacteroides thetaiotaomicron* have the capacity to induce severe ulcerative disease (27).

1.3.2. Consortium networks formed between microorganisms: the biological system according to the law of system completeness

Possible microbial consortia or networks were explored to shed light on the ecology of these microorganisms and their behaviour in the host. Networks between species, genera and families were analysed and the complexity indexes of the consortia formed by the most abundant species (over 30-35) were calculated. The results, shown in Supplementary Figure 3 and Supplementary Table 5, indicate that the bacterial complexity in the control cohort is greater than in IBD patients, which agrees with the higher microbial biodiversity observed in that group. The least complexity was observed in the networks generated at disease onset in CD and UC patients, and although it increased throughout the treatment period, it remained lower than in the control cohort at 6M. Nevertheless, this result should be treated with caution due to the small number of samples.

1.4. Supplementary Figures and Tables

In this section the supplementary tables and figures to the main article will be indicated.

1.4.1. Supplementary Figures

Supplementary Figure 1. In A to D are shown, respectively, the circular pie charts of percentages for the following groups: faecal samples from ulcerative colitis (FE-UC) and Crohn's disease (FE-CD) patients, and oral samples from UC (OR-UC) and CD patients. HC (or CO), DE, 3M and 6M refer to the control cohort, and sampling points at disease onset, 3 months and 6 months. For simplification, only species with a relative frequency > 2 % and genera and families with a relative frequency > 1 % are represented.

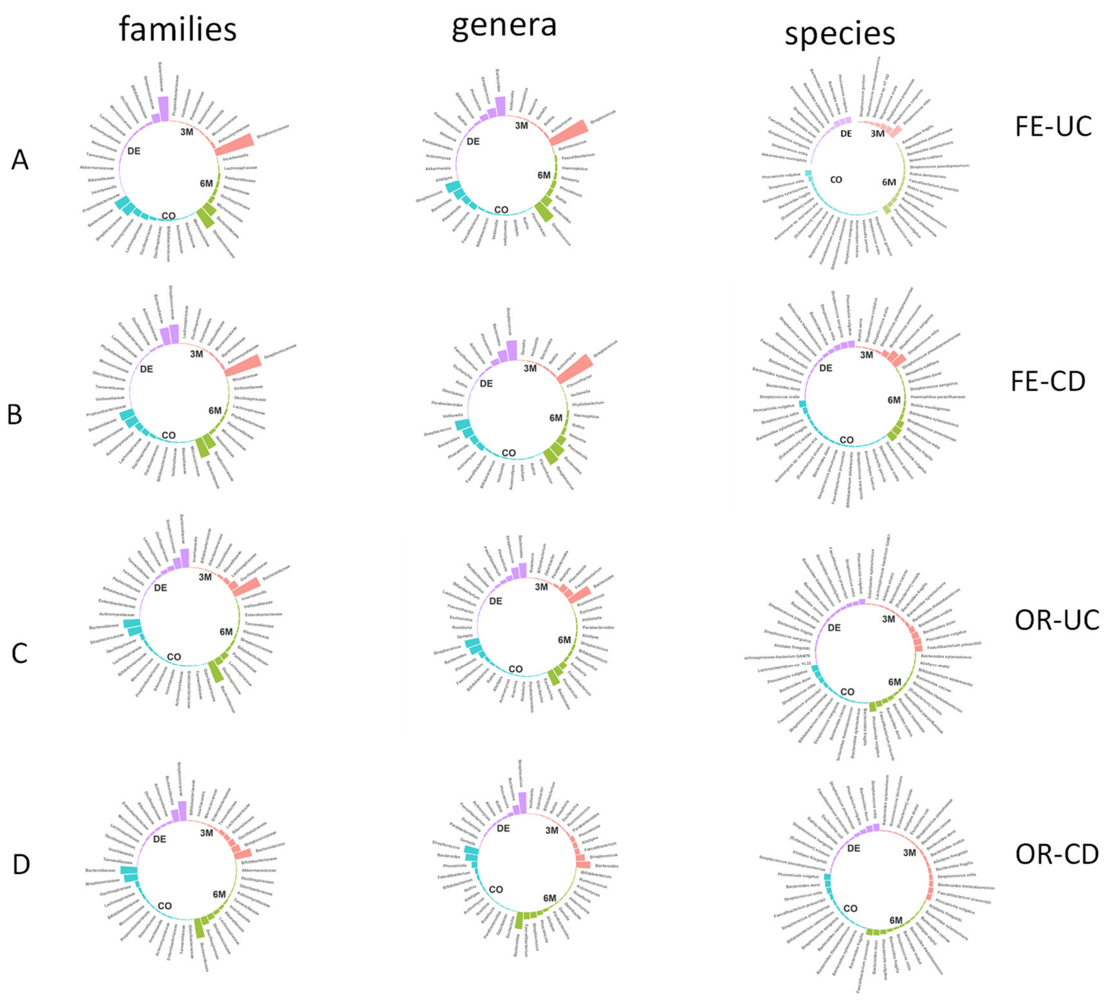

Some examples of bacterial species consortia formed in the samples during the clinical evolution of the patients using two different grouping methods (walk trap and Girvan Newman communities). In A, B and C are presented two examples. In A, several well-formed groups connected (faecal control and oral control) for: located on the left HC-FE and on the right HC-OR. In B, several well-formed groups connected for DE-CD-FE (left), DE-UC-FE (right). In C, groups connected for 3M-CD-FE (left), 3M-UC-FE (right). And finally, in D, groups connected for 6M-CD-FE (left side) and 6M-UC-FE (right side).

Supplementary Figure 2. Graphical summary of the binomial proportion tests applied to bacterial species.

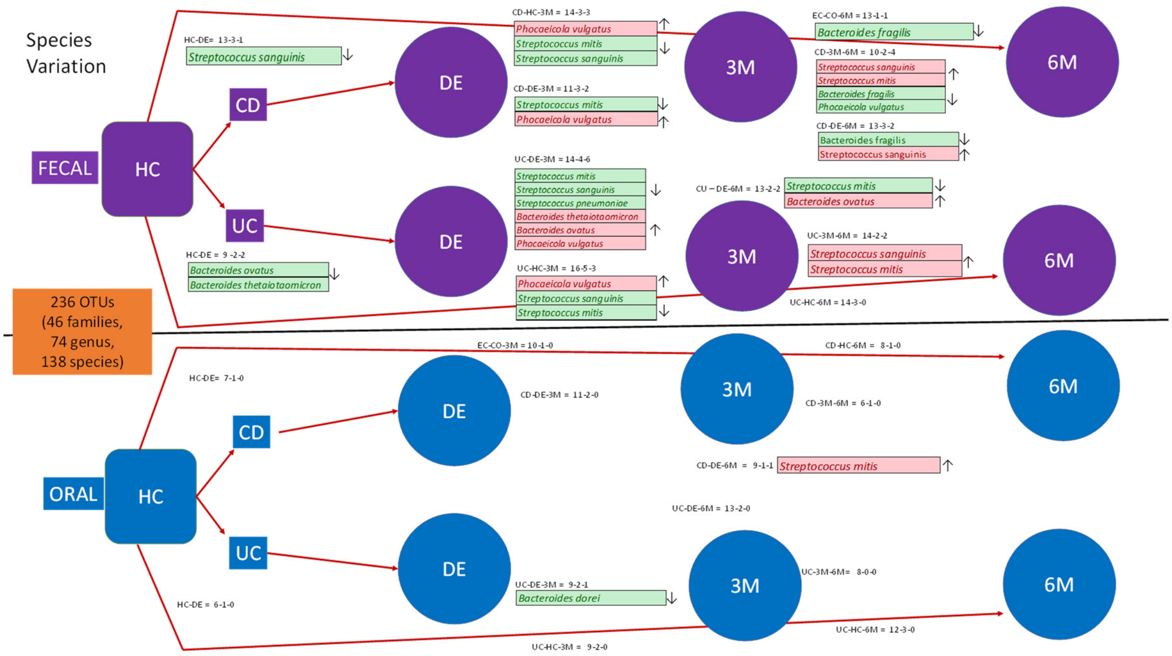

The formulas indicate the number of statistically different families, genera, and species between stages of the experimental design (e.g., FECAL-CD HC-DE 13-3-1: 13 families, 3 genera and 1 species are statistically different between the healthy control (HC) samples and faecal samples in the Crohn's disease (CD) patients at disease onset (DE))

1.4.2. Supplementary Tables

Supplementary Table 1. Result of the binomial test applied to percentages of bacterial species detected in the different groups.

Species	FE-HC	FE-UC-3M	FE-UC-6M	FE-UC-DE	FE-CD-3M	FE-CD-6M	FE-CD-DE	OR-HC	OR-UC-3M	OR-UC-6M	OR-UC-DE	OR-CD-3M	OR-CD-3M	OR-CD-6M
[Eubacterium] rectale	4.83	0.00	0.30	0.84	0.14	0.01	0.11	1.55	3.29	4.17	1.75	2.32	1.76	0.76
[Eubacterium] siraeum	4.55	0.00	0.02	0.02	0.02	0.00	0.07	0.11	0.50	0.09	0.56	0.20	1.92	3.11
[Ruminococcus] torques	0.51	0.00	0.27	0.19	0.16	0.02	0.85	0.22	0.46	0.39	0.21	0.38	0.38	0.12
Abiotrophia defectiva	0.00	0.01	0.00	0.01	0.02	0.00	0.04	0.02	0.00	0.00	0.01	0.01	0.01	0.01
Acidaminococcus intestini	0.00	0.00	0.00	0.00	0.00	0.00	0.45	0.00	0.00	0.00	0.00	0.01	0.00	0.00
Actinomyces naeslundii	0.45	0.74	0.15	0.34	0.88	0.27	0.72	0.27	0.00	0.00	0.18	0.13	0.03	0.46
Actinomyces oris	0.64	1.01	0.19	0.49	1.21	0.37	1.04	0.39	0.00	0.00	0.25	0.18	0.05	0.65
Actinomyces pacaensis	0.01	0.01	0.00	0.01	0.04	0.00	0.01	0.02	0.00	0.00	0.01	0.01	0.01	0.04
Actinomyces radicidentis	0.17	0.02	0.00	0.02	0.01	0.00	0.01	0.01	0.00	0.00	0.00	0.00	0.01	0.06
Actinomyces sp. Chiba101	0.11	0.02	0.00	0.02	0.01	0.00	0.01	0.01	0.00	0.00	0.00	0.00	0.01	0.04
Actinomyces sp. oral taxon 414	4.60	0.73	0.00	0.13	0.20	0.00	0.13	0.28	0.00	0.00	0.02	0.03	0.43	0.78
Actinomyces sp. oral taxon 897	0.21	0.03	0.00	0.06	0.02	0.00	0.01	0.02	0.00	0.00	0.00	0.01	0.73	0.05
Adlercreutzia equolifaciens	0.00	0.00	0.00	0.02	0.00	0.00	0.00	0.01	0.03	0.00	0.01	0.01	0.03	0.00
Aggregatibacter aphrophilus	0.01	0.13	0.13	0.04	0.01	0.29	0.02	0.01	0.00	0.03	0.09	0.00	0.01	0.00
Akkermansia muciniphila	0.28	0.00	0.00	2.09	1.17	0.00	0.77	0.00	0.41	0.00	0.02	0.98	1.77	0.43
Alistipes finegoldii	0.36	0.00	0.04	0.49	0.28	0.00	0.36	1.61	1.74	0.63	3.33	4.70	2.03	2.66

Species	FE-HC	FE-UC-3M	FE-UC-6M	FE-UC-DE	FE-CD-3M	FE-CD-6M	FE-CD-DE	OR-HC	OR-UC-3M	OR-UC-6M	OR-UC-DE	OR-CD-3M	OR-CD-3M	OR-CD-6M
Alistipes shahii	1.38	0.00	0.69	0.74	0.43	0.17	0.31	1.23	2.86	2.31	1.58	2.40	3.13	1.37
Anaerobutyricum hallii	0.09	0.00	0.19	0.06	0.01	0.01	0.03	0.11	0.18	0.15	0.07	0.13	0.10	0.06
Anaerostipes hadrus	2.28	0.00	0.40	0.10	0.10	0.27	0.04	0.39	0.55	0.67	0.20	0.49	0.47	0.22
Arachnia propionica	0.23	0.94	0.00	0.71	0.59	0.00	1.16	1.88	0.00	0.00	0.28	0.32	0.37	0.29
Bacteroides caccae	0.65	0.00	1.96	0.64	0.41	0.03	3.08	2.90	3.06	3.09	4.68	2.88	2.51	1.08
Bacteroides caecimuris	0.72	0.01	0.27	1.35	0.06	0.63	0.33	0.41	0.63	0.41	0.49	0.51	0.63	0.22
Bacteroides cellulosilyticus	0.16	0.00	0.13	0.72	0.62	0.08	0.58	0.35	0.39	0.47	5.79	0.73	1.44	0.30
Bacteroides dorei	3.89	0.09	5.08	2.81	1.10	2.32	2.16	11.50	13.97	8.73	3.61	4.25	11.71	12.79
Bacteroides fragilis	4.92	0.02	2.02	0.95	0.33	16.90	0.56	2.07	3.34	1.84	3.35	5.87	5.79	1.57
Bacteroides helcogenes	0.03	0.00	0.02	0.09	0.01	0.07	0.06	0.06	0.11	0.03	0.08	0.08	0.11	0.09
Bacteroides heparinolyticus	0.03	0.00	0.04	0.04	0.00	0.11	0.07	0.06	0.06	0.02	0.04	0.05	0.07	0.03
Bacteroides ovatus	1.53	0.03	1.53	13.21	0.32	1.31	8.06	1.93	11.38	7.94	4.70	4.55	4.16	1.06
Bacteroides thetaiotaomicron	0.83	0.02	6.18	13.16	0.16	0.44	6.33	2.59	5.99	3.13	5.29	8.12	3.85	1.13
Bacteroides uniformis	0.04	0.00	0.02	0.31	0.01	0.05	0.06	0.17	0.14	0.03	0.11	0.10	0.23	0.06
Bacteroides xylanisolvens	5.09	0.09	2.32	5.77	0.18	4.74	2.51	2.14	4.14	2.27	1.95	2.09	2.33	1.24
Barnesiella viscericola	0.09	0.00	0.03	0.07	0.01	0.01	0.03	0.09	0.10	0.05	0.05	0.09	0.11	0.07
Bifidobacterium adolescentis	2.43	0.00	0.00	4.77	0.00	0.01	0.00	0.06	0.01	2.44	1.34	0.94	0.60	0.01
Bifidobacterium angulatum	0.00	0.00	0.00	0.02	0.00	0.00	0.00	0.02	0.00	0.46	0.01	0.00	0.01	0.00

Species	FE-HC	FE-UC-3M	FE-UC-6M	FE-UC-DE	FE-CD-3M	FE-CD-6M	FE-CD-DE	OR-HC	OR-UC-3M	OR-UC-6M	OR-UC-DE	OR-CD-3M	OR-CD-3M	OR-CD-6M
Bifidobacterium bifidum	0.00	0.00	0.00	0.21	0.00	0.00	0.00	0.02	0.00	0.06	0.08	0.02	0.02	0.00
Bifidobacterium breve	0.00	0.00	0.01	0.03	0.00	0.00	0.00	0.04	0.02	0.09	0.03	0.01	0.02	0.01
Bifidobacterium catenulatum	0.40	0.00	0.00	0.07	0.00	0.48	0.01	3.36	0.11	0.13	0.11	0.04	0.08	0.01
Bifidobacterium longum	0.74	0.00	0.00	1.09	0.03	0.05	0.04	0.88	1.28	1.16	0.22	0.68	0.49	0.23
Bifidobacterium pseudocatenulatum	0.08	0.00	0.00	0.02	0.01	0.03	0.02	0.45	0.30	0.65	0.33	0.13	0.14	0.05
Blautia argi	0.03	0.00	0.02	0.05	0.00	0.06	0.02	0.01	0.03	0.03	0.04	0.03	0.03	0.02
Blautia hansenii	0.29	0.00	0.04	0.02	0.01	0.57	0.03	0.04	0.05	0.04	0.06	0.12	0.11	0.03
Blautia obeum	0.24	0.00	0.20	0.10	0.05	0.03	0.12	0.16	0.31	0.23	0.14	0.24	0.29	0.09
butyrate-producing bacterium SM4/1	0.02	0.00	0.02	0.02	0.01	0.03	0.02	0.02	0.03	0.02	0.03	0.08	0.16	0.11
butyrate-producing bacterium SS3/4	0.16	0.00	0.37	0.36	0.09	0.01	0.25	1.04	0.57	1.63	0.73	0.49	1.07	0.25
Campylobacter concisus	0.01	0.06	0.09	0.01	0.01	0.07	0.00	0.00	0.00	0.31	0.00	0.00	0.01	0.00
Capnocytophaga sputigena	0.01	0.21	0.20	0.09	0.01	0.16	0.01	0.01	0.00	0.00	0.07	0.00	0.00	0.00
Citrobacter freundii	0.00	0.00	0.00	0.00	0.00	0.00	0.00	0.07	0.01	0.01	0.00	0.00	0.00	0.05
Clostridiales bacterium CCNA10	0.21	0.00	0.46	0.33	0.08	0.11	0.18	0.80	0.36	0.18	0.37	0.28	0.41	0.17
Clostridioides difficile	0.10	0.00	0.05	0.06	0.04	0.09	0.08	0.08	0.13	0.12	0.09	0.13	0.18	0.07
Clostridium perfringens	0.00	0.00	0.00	0.01	0.00	0.00	0.00	0.00	0.00	0.22	0.02	0.01	0.00	0.00
Collinsella aerofaciens	0.14	0.00	0.00	0.08	0.12	0.00	0.08	0.30	0.43	0.33	0.31	0.32	0.38	0.49
Coprococcus catus	0.13	0.00	0.06	0.06	0.04	0.00	0.07	0.10	0.19	0.20	0.09	0.17	0.22	0.05

Species	FE-HC	FE-UC-3M	FE-UC-6M	FE-UC-DE	FE-CD-3M	FE-CD-6M	FE-CD-DE	OR-HC	OR-UC-3M	OR-UC-6M	OR-UC-DE	OR-CD-3M	OR-CD-3M	OR-CD-6M
Coprococcus sp. ART55/1	0.42	0.00	0.03	0.01	0.02	0.00	0.02	0.05	0.06	0.07	0.09	0.07	0.48	0.03
Cutibacterium acnes	0.01	0.03	0.00	0.02	0.04	0.24	0.08	0.03	0.00	0.00	0.00	0.01	0.00	0.02
Desulfovibrio piger	0.00	0.00	0.00	0.00	0.00	0.00	0.11	0.00	0.05	0.01	0.00	0.07	0.17	0.06
Eggerthella lenta	0.02	0.01	0.07	0.03	0.00	0.01	0.00	0.04	0.05	0.01	0.01	0.11	0.02	0.04
Enterobacter cloacae	0.00	0.00	0.00	0.11	0.00	0.00	0.00	0.00	0.00	0.01	0.00	0.00	0.01	0.00
Enterocloster bolteae	0.26	0.00	0.34	0.45	0.04	0.65	0.22	0.12	0.29	0.10	0.36	0.81	0.27	0.19
Enterococcus faecium	0.00	0.00	0.01	0.00	0.00	0.00	0.00	0.08	0.01	0.04	0.01	0.00	0.02	0.00
Erysipelotrichaceae bacterium GAM147	0.23	0.00	0.01	0.05	0.04	0.01	0.01	0.15	0.26	0.29	0.15	0.31	0.29	0.10
Escherichia coli	0.14	0.09	0.00	0.51	0.35	0.16	1.98	1.25	0.40	1.52	1.44	2.62	0.23	3.89
Faecalibacterium prausnitzii	3.08	0.01	4.68	2.76	0.82	0.07	3.15	6.62	15.75	12.13	7.80	9.83	13.12	3.93
Faecalitalea cylindroides	0.10	0.00	0.03	0.03	0.02	0.01	0.05	0.06	0.09	0.08	0.05	0.08	0.14	0.04
Flavonifractor plautii	1.27	0.00	0.24	0.52	0.03	1.40	0.20	0.77	0.75	0.43	1.96	0.87	0.99	0.48
Fusobacterium nucleatum	0.01	0.25	0.20	0.05	0.14	0.26	0.02	0.01	0.00	0.12	0.06	0.02	0.01	0.04
Fusobacterium periodonticum	0.02	0.09	0.61	0.07	0.01	0.38	0.01	0.01	0.00	0.00	0.14	0.03	0.03	0.00
Gemella morbillorum	0.26	1.40	0.21	0.47	0.80	0.21	0.24	0.59	0.00	0.00	0.65	0.45	1.73	0.73
Haemophilus haemolyticus	0.04	0.17	0.99	0.13	0.01	1.00	0.12	0.02	0.00	0.19	0.07	0.00	0.02	0.03
Haemophilus influenzae	0.01	0.07	0.31	0.05	0.01	0.30	0.04	0.01	0.00	0.08	0.05	0.00	0.01	0.01
Haemophilus parainfluenzae	0.24	1.78	2.19	0.70	0.56	3.08	0.49	0.30	0.07	4.56	1.85	0.18	0.25	0.03

Species	FE-HC	FE-UC-3M	FE-UC-6M	FE-UC-DE	FE-CD-3M	FE-CD-6M	FE-CD-DE	OR-HC	OR-UC-3M	OR-UC-6M	OR-UC-DE	OR-CD-3M	OR-CD-3M	OR-CD-6M
Haemophilus pittmaniae	0.01	0.06	0.07	0.02	0.00	0.09	0.01	0.01	0.00	0.16	0.05	0.00	0.01	0.00
Haemophilus sp. oral taxon 036	0.02	0.06	0.70	0.07	0.01	0.38	0.06	0.01	0.00	0.03	0.02	0.00	0.00	0.01
Hafnia paralvei	0.00	0.00	0.00	0.00	0.00	0.00	0.00	0.30	0.00	0.00	0.00	0.00	0.00	0.00
Intestinimonas butyriciproducens	0.09	0.00	0.32	0.04	0.09	0.01	0.11	0.09	0.18	0.12	0.14	0.13	0.29	0.10
Lachnoclostridium phocaeense	0.00	0.00	0.01	0.00	0.00	0.00	0.00	0.01	0.02	0.02	0.05	0.02	0.04	0.01
Lachnoclostridium sp. YL32	0.05	0.00	0.04	0.13	0.01	0.14	0.04	0.02	0.04	0.03	2.02	0.48	0.05	0.03
Lachnospira eligens	0.36	0.00	0.01	0.14	0.04	0.00	0.02	0.41	0.57	0.49	0.13	0.59	0.36	0.33
Lachnospiraceae bacterium Choco86	0.09	0.00	0.06	0.03	0.01	0.01	0.04	0.05	0.09	0.11	0.07	0.07	0.11	0.03
Lachnospiraceae bacterium GAM79	1.81	0.00	0.06	0.19	0.23	0.03	0.52	0.72	2.08	0.61	2.35	0.69	1.24	0.10
Lacrimispora saccharolytica	0.05	0.00	0.02	0.03	0.01	0.05	0.03	0.06	0.09	0.07	0.07	0.19	0.42	0.32
Lancefieldella parvula	0.12	0.02	0.00	0.00	0.04	0.00	0.01	0.01	0.00	0.00	0.00	0.00	0.14	0.01
Massilistercora timonensis	0.04	0.00	0.00	0.01	0.01	0.01	0.04	0.03	0.04	0.03	0.03	0.05	0.05	0.02
Megamonas hypermegale	0.00	0.00	0.00	0.00	0.08	0.00	1.10	0.03	0.00	0.00	0.00	0.00	0.02	0.00
Methanobrevibacter smithii	0.00	0.00	0.00	0.00	0.00	0.00	0.00	0.00	0.00	0.00	0.00	0.01	0.00	0.09
Neisseria elongata	0.03	0.36	0.30	0.21	0.03	0.51	0.02	0.01	0.00	0.00	0.10	0.00	0.00	0.01
Neisseria mucosa	0.07	0.51	0.59	0.57	0.05	1.29	0.03	0.01	0.00	0.00	0.11	0.00	0.00	0.05
Neisseria sicca	0.09	0.58	0.70	0.71	0.05	1.37	0.03	0.01	0.00	0.00	0.12	0.00	0.00	0.08

Species	FE-HC	FE-UC-3M	FE-UC-6M	FE-UC-DE	FE-CD-3M	FE-CD-6M	FE-CD-DE	OR-HC	OR-UC-3M	OR-UC-6M	OR-UC-DE	OR-CD-3M	OR-CD-3M	OR-CD-6M
Neisseria subflava	0.22	0.47	3.11	0.31	0.15	2.30	0.02	0.09	0.00	0.00	0.05	0.00	0.06	0.01
Odoribacter splanchnicus	0.36	0.01	0.60	0.27	0.09	0.04	1.67	1.54	2.05	0.47	1.06	1.24	1.96	0.90
Olsenella sp. oral taxon 807	0.69	0.34	0.00	0.03	0.30	0.00	0.09	0.10	0.00	0.00	0.00	0.01	0.76	0.03
Ornithobacterium rhinotracheale	0.10	0.00	0.02	0.06	0.01	0.14	0.02	0.05	0.07	0.07	0.04	0.05	0.08	0.05
Oscillibacter sp. PEA192	0.75	0.00	0.18	0.54	0.10	0.57	0.16	0.42	0.56	0.36	0.54	0.62	0.62	0.20
Parabacteroides distasonis	0.11	0.00	0.02	0.61	0.03	0.05	0.40	0.29	0.46	0.70	0.71	0.89	0.66	0.36
Parabacteroides sp. CT06	0.12	0.00	0.30	0.77	0.02	0.04	0.42	0.73	0.73	0.79	0.81	1.10	0.68	0.67
Pauljensenia hongkongensis	0.55	0.28	0.00	0.10	0.39	0.00	0.02	0.08	0.00	0.00	0.04	0.02	0.05	0.13
Phocaeicola salanitronis	0.06	0.00	0.01	0.08	0.01	0.10	0.11	0.12	0.14	0.07	0.05	0.08	0.13	0.07
Phocaeicola vulgatus	14.31	0.30	9.70	13.99	0.54	18.29	13.99	13.36	15.53	19.48	11.40	9.93	9.57	8.70
Phyllobacterium zundukense	0.01	0.06	0.00	0.03	0.10	0.43	0.15	0.05	0.00	0.00	0.01	0.02	0.01	0.02
Porphyromonas asaccharolytica	0.00	0.00	0.00	0.00	0.00	0.00	0.00	0.01	0.70	0.00	0.02	0.00	0.00	0.00
Prevotella melaninogenica	0.06	0.30	0.55	0.11	0.07	0.35	0.00	0.02	0.02	0.00	0.03	0.00	0.05	0.00
Proteus vulgaris	0.00	0.00	0.00	0.00	0.00	0.00	0.00	0.00	0.00	0.00	0.00	0.14	0.00	0.00
Romboutsia ilealis	0.00	0.00	0.34	0.00	0.00	0.00	0.00	0.03	0.02	0.02	0.01	0.01	0.04	0.00
Roseburia hominis	0.13	0.00	0.04	0.06	0.13	0.02	0.26	0.32	0.34	0.66	0.69	0.40	0.27	0.20
Roseburia intestinalis	0.53	0.00	0.06	0.13	0.08	0.03	0.11	1.46	0.66	4.93	0.46	1.82	1.37	0.50

Species	FE-HC	FE-UC-3M	FE-UC-6M	FE-UC-DE	FE-CD-3M	FE-CD-6M	FE-CD-DE	OR-HC	OR-UC-3M	OR-UC-6M	OR-UC-DE	OR-CD-3M	OR-CD-3M	OR-CD-6M
Rothia aeria	0.33	0.85	1.24	1.40	2.21	0.84	0.43	1.02	0.00	0.00	0.29	0.07	0.05	0.87
Rothia dentocariosa	0.35	1.28	3.77	1.09	0.48	0.87	0.37	0.34	0.00	0.00	0.38	0.09	0.13	0.24
Rothia mucilaginosa	1.20	1.84	4.84	1.46	1.62	4.13	0.79	1.91	0.00	0.08	0.02	1.82	0.60	3.82
Ruminococcus bicirculans	0.28	0.00	0.06	0.32	0.04	0.00	0.00	0.42	0.11	0.23	0.46	2.25	1.09	0.10
Ruminococcus champanellensis	0.00	0.00	0.00	0.01	0.00	0.00	0.00	0.01	0.01	0.01	0.01	0.00	0.11	0.00
Ruminococcus sp. SR1/5	0.17	0.00	1.58	0.12	0.02	0.07	0.05	0.20	0.25	0.82	0.19	0.30	0.31	0.13
Schaalia meyeri	0.08	0.08	0.00	0.02	0.12	0.00	0.05	0.03	0.00	0.00	0.02	0.01	0.04	0.09
Streptococcus anginosus	0.50	0.02	0.00	0.02	0.08	0.00	0.03	0.02	0.00	0.00	0.01	0.01	0.73	0.04
Streptococcus australis	0.05	0.30	0.00	0.19	0.21	0.00	0.23	0.11	0.00	0.00	0.03	0.06	0.02	0.95
Streptococcus constellatus	0.01	0.01	0.00	0.01	0.02	0.00	0.01	0.01	0.00	0.00	0.00	0.00	0.11	0.01
Streptococcus cristatus	1.06	0.72	0.28	0.97	2.30	0.61	0.77	1.81	0.00	0.00	0.23	0.70	0.36	1.49
Streptococcus dysgalactiae	0.01	0.02	0.00	0.00	0.05	0.00	0.05	0.00	0.00	0.00	0.00	0.00	0.00	0.01
Streptococcus equinus	0.03	0.02	0.00	0.03	0.02	0.00	0.01	0.04	0.00	0.00	0.00	0.04	0.00	0.08
Streptococcus gordonii	2.02	2.29	0.25	1.44	1.58	0.43	4.10	0.61	0.00	0.02	0.58	0.46	0.47	1.70
Streptococcus ilei	0.09	0.14	0.00	0.08	0.20	0.00	0.14	0.07	0.00	0.00	0.02	0.04	0.01	0.53
Streptococcus intermedius	0.08	0.08	0.00	0.40	0.24	0.00	0.11	0.27	0.00	0.00	0.07	0.04	0.05	0.08
Streptococcus mitis	8.93	28.80	18.42	3.94	28.81	13.31	11.03	9.44	0.00	0.00	8.02	6.98	4.37	15.18
Streptococcus mutans	0.00	0.00	0.00	0.05	0.02	0.00	0.01	0.05	0.00	0.00	0.00	0.01	0.01	0.00
Streptococcus oralis	2.11	8.21	1.27	2.22	2.80	1.40	2.04	1.39	0.00	0.00	1.18	0.79	0.34	1.78

Species	FE-HC	FE-UC-3M	FE-UC-6M	FE-UC-DE	FE-CD-3M	FE-CD-6M	FE-CD-DE	OR-HC	OR-UC-3M	OR-UC-6M	OR-UC-DE	OR-CD-3M	OR-CD-3M	OR-CD-6M
Streptococcus parasanguinis	0.13	0.41	0.26	0.31	0.18	0.24	0.16	0.39	0.01	1.13	0.05	0.20	0.03	0.33
Streptococcus pneumoniae	3.55	12.13	8.88	1.60	12.29	5.86	4.30	4.22	0.00	0.00	3.49	2.84	1.89	6.61
Streptococcus pseudopneumoniae	1.28	4.73	3.29	0.55	4.82	2.19	1.64	1.55	0.00	0.00	1.31	1.05	0.70	2.63
Streptococcus salivarius	0.39	0.24	0.36	0.25	0.17	0.26	0.17	0.21	0.04	1.67	0.04	0.25	0.05	0.66
Streptococcus sanguinis	2.32	14.58	0.90	2.55	22.36	2.38	12.72	3.34	0.00	0.00	3.35	0.72	0.59	3.39
Streptococcus sp. A12	0.07	0.80	0.47	0.20	0.42	0.16	0.60	0.26	0.01	0.03	0.09	0.10	0.07	1.54
Streptococcus sp. FDAARGOS_192	0.03	0.02	0.00	0.01	0.01	0.00	0.02	0.02	0.00	0.00	0.00	0.01	0.00	0.07
Streptococcus sp. NPS 308	0.40	1.14	0.39	0.42	0.59	0.66	0.45	0.35	0.00	0.00	0.24	0.17	0.11	0.38
Streptococcus sp. oral taxon 064	0.35	1.82	0.19	0.39	0.46	0.22	0.31	0.21	0.00	0.00	0.20	0.13	0.05	0.32
Streptococcus sp. oral taxon 431	0.25	0.55	1.28	0.31	0.63	0.86	0.50	0.35	0.00	0.01	0.18	0.12	0.11	0.33
Streptococcus sp. VT 162	0.62	5.78	0.30	0.76	0.77	0.35	0.48	0.40	0.00	0.00	0.31	0.22	0.08	0.52
Streptococcus thermophilus	0.22	0.04	0.23	0.04	0.11	0.02	0.04	0.07	0.07	0.04	0.03	0.03	0.04	0.54
Veillonella atypica	0.24	0.13	0.16	0.08	0.59	0.09	0.17	0.03	0.01	0.78	0.02	0.38	0.04	0.02
Veillonella parvula	2.24	1.55	0.36	0.65	1.21	1.21	1.09	0.39	0.04	1.51	0.47	0.71	0.09	0.47
Victivallales bacterium CCUG 44730	0.00	0.00	0.00	0.00	0.00	0.00	0.00	0.01	0.05	0.02	0.05	0.01	0.07	0.01

Supplementary Table 2. Result of the binomial test applied to percentages of bacterial genera detected in the different groups.

GENUS	FE-HC	FE-UC-3M	FE-UC-6M	FE-UC-DE	FE-CD-3M	FE-CD-6M	FE-CD-DE	OR-HC	OR-UC-3M	OR-UC-6M	OR-UC-DE	OR-CD-3M	OR-CD-3M	OR-CD-6M
Abiotrophia	0.00	0.01	0.00	0.01	0.02	0.00	0.04	0.01	0.00	0.00	0.01	0.01	0.01	0.01
Acidaminococcus	0.00	0.00	0.00	0.00	0.00	0.00	0.38	0.00	0.00	0.00	0.00	0.01	0.00	0.00
Actinomyces	11.01	4.23	0.66	2.06	4.18	0.96	4.00	2.01	0.00	0.01	0.98	0.83	1.60	4.12
Adlercreutzia	0.00	0.00	0.00	0.02	0.00	0.00	0.00	0.01	0.03	0.00	0.01	0.01	0.02	0.00
Aggregatibacter	0.01	0.10	0.11	0.03	0.01	0.24	0.02	0.01	0.00	0.03	0.08	0.00	0.01	0.00
Akkermansia	0.50	0.00	0.00	1.84	0.93	0.00	0.65	0.00	0.60	0.01	0.02	0.88	1.64	0.41
Alistipes	1.90	0.00	0.66	1.25	0.66	0.15	0.61	2.68	5.15	3.03	4.94	8.81	5.67	4.19
Anaerobutyricum	0.09	0.00	0.16	0.05	0.01	0.01	0.02	0.10	0.18	0.15	0.07	0.11	0.09	0.05
Anaerostipes	2.10	0.00	0.35	0.09	0.08	0.22	0.03	0.34	0.53	0.65	0.18	0.43	0.43	0.19
Arachnia	0.21	0.71	0.00	0.61	0.46	0.00	0.98	1.65	0.00	0.00	0.26	0.28	0.35	0.25
Atopobium	0.00	0.01	0.00	0.00	0.02	0.00	0.01	0.00	0.00	0.00	0.00	0.00	0.09	0.00
Bacteroides	20.26	0.25	18.55	39.46	3.00	26.18	24.01	24.69	47.69	31.66	30.75	28.89	34.49	19.27
Barnesiella	0.08	0.00	0.02	0.07	0.01	0.01	0.03	0.10	0.12	0.07	0.06	0.09	0.14	0.06
Bifidobacterium	3.55	0.00	0.01	5.57	0.03	0.54	0.07	4.81	1.79	5.10	2.05	1.69	1.36	0.33
Blautia	0.68	0.00	0.28	0.19	0.07	0.65	0.21	0.25	0.52	0.40	0.32	0.49	0.53	0.17
Campylobacter	0.01	0.04	0.08	0.01	0.01	0.06	0.00	0.00	0.00	0.30	0.00	0.00	0.01	0.00
Capnocytophaga	0.01	0.16	0.17	0.08	0.00	0.13	0.01	0.01	0.00	0.00	0.07	0.00	0.00	0.00
Citrobacte	0.00	0.00	0.00	0.00	0.00	0.00	0.00	0.06	0.01	0.01	0.00	0.04	0.00	0.03
Citrobacter	0.00	0.00	0.00	0.00	0.00	0.00	0.00	0.06	0.01	0.01	0.00	0.00	0.00	0.04
Clostridioides	0.09	0.00	0.05	0.05	0.03	0.07	0.07	0.07	0.13	0.12	0.08	0.11	0.16	0.06

GENUS	FE-HC	FE-UC-3M	FE-UC-6M	FE-UC-DE	FE-CD-3M	FE-CD-6M	FE-CD-DE	OR-HC	OR-UC-3M	OR-UC-6M	OR-UC-DE	OR-CD-3M	OR-CD-3M	OR-CD-6M
Clostridium	0.00	0.00	0.00	0.01	0.00	0.00	0.00	0.00	0.00	0.22	0.02	0.01	0.00	0.00
Collinsella	0.16	0.00	0.00	0.08	0.11	0.00	0.08	0.29	0.46	0.35	0.32	0.31	0.40	0.46
Coprococcus	0.53	0.00	0.07	0.07	0.06	0.01	0.09	0.13	0.26	0.26	0.17	0.23	0.66	0.08
Corynebacterium	0.01	0.00	0.00	0.01	0.03	0.00	0.01	0.02	0.00	0.00	0.00	0.01	0.00	0.02
Cutibacterium	0.01	0.02	0.00	0.02	0.03	0.20	0.07	0.02	0.00	0.00	0.00	0.01	0.00	0.01
Desulfovibrio	0.00	0.00	0.00	0.00	0.00	0.00	0.09	0.00	0.05	0.01	0.00	0.06	0.15	0.05
Eggerthella	0.02	0.01	0.06	0.02	0.00	0.01	0.00	0.03	0.04	0.01	0.01	0.10	0.02	0.03
Ensifer	0.00	0.01	0.00	0.01	0.01	0.08	0.02	0.01	0.00	0.00	0.00	0.00	0.00	0.00
Enterobacter	0.00	0.00	0.00	0.12	0.00	0.00	0.00	0.01	0.00	0.01	0.00	0.01	0.13	0.00
Enterocloster	0.24	0.00	0.29	0.39	0.03	0.53	0.19	0.10	0.28	0.10	0.33	0.71	0.25	0.16
Enterococcus	0.01	0.02	0.01	0.01	0.02	0.01	0.02	0.08	0.01	0.03	0.01	0.01	0.03	0.02
Escherichia	0.13	0.07	0.00	0.45	0.28	0.13	1.67	1.11	0.39	1.50	1.33	2.32	0.21	3.32
Eubacterium	0.10	0.00	0.01	0.04	0.01	0.00	0.02	0.04	0.09	0.09	0.03	0.08	0.04	0.03
Faecalibacterium	3.58	0.01	4.19	2.61	0.71	0.07	3.29	6.25	16.38	13.20	7.58	9.56	14.27	3.78
Faecalitalea	0.09	0.00	0.03	0.02	0.01	0.00	0.04	0.05	0.09	0.07	0.05	0.07	0.13	0.04
Flavonifractor	1.21	0.00	0.21	0.46	0.04	1.18	0.19	0.71	0.78	0.44	1.84	0.81	0.98	0.44
Fusobacterium	0.03	0.26	0.69	0.10	0.12	0.53	0.03	0.01	0.00	0.12	0.19	0.04	0.04	0.04
Gemella	0.33	2.45	0.18	0.48	1.02	0.18	0.38	0.95	0.00	0.00	1.04	0.84	2.16	1.17
Haemophilus	0.33	1.81	4.31	0.96	0.48	4.64	0.70	0.34	0.08	5.35	2.08	0.18	0.30	0.09
Hafnia	0.00	0.00	0.00	0.00	0.00	0.00	0.00	0.26	0.00	0.00	0.00	0.00	0.00	0.00
Intestinimonas	0.12	0.00	0.28	0.05	0.10	0.02	0.12	0.11	0.23	0.15	0.15	0.15	0.35	0.11

GENUS	FE-HC	FE-UC-3M	FE-UC-6M	FE-UC-DE	FE-CD-3M	FE-CD-6M	FE-CD-DE	OR-HC	OR-UC-3M	OR-UC-6M	OR-UC-DE	OR-CD-3M	OR-CD-3M	OR-CD-6M
Klebsiella	0.00	0.00	0.00	0.02	0.24	0.03	0.01	0.13	0.02	0.09	0.00	0.00	0.01	0.01
Lachnoclostridium	0.15	0.00	0.07	0.24	0.02	0.30	0.08	0.06	0.13	0.11	2.05	0.55	0.17	0.08
Lachnospira	0.33	0.00	0.01	0.12	0.03	0.00	0.02	0.36	0.56	0.47	0.12	0.51	0.34	0.28
Lacrimispora	0.04	0.00	0.01	0.02	0.01	0.04	0.03	0.05	0.08	0.07	0.07	0.17	0.39	0.27
Lancefieldella	0.11	0.01	0.00	0.00	0.03	0.00	0.01	0.01	0.00	0.00	0.00	0.00	0.13	0.01
Massilistercora	0.04	0.00	0.00	0.01	0.01	0.01	0.03	0.02	0.04	0.03	0.03	0.05	0.05	0.02
Mediterraneibacter	0.47	0.00	0.23	0.17	0.12	0.02	0.71	0.19	0.45	0.38	0.19	0.34	0.35	0.10
Megamonas	0.00	0.00	0.00	0.00	0.06	0.00	0.92	0.02	0.00	0.00	0.00	0.00	0.02	0.00
Mesorhizobium	0.00	0.02	0.00	0.01	0.03	0.16	0.05	0.02	0.00	0.00	0.00	0.01	0.00	0.01
Methanobrevibacter	0.00	0.00	0.00	0.00	0.00	0.00	0.00	0.00	0.00	0.00	0.00	0.01	0.00	0.08
Neisseria	0.53	2.22	5.24	2.48	0.31	6.71	0.12	0.14	0.00	0.01	0.53	0.00	0.07	0.21
Odoribacter	0.33	0.01	0.51	0.23	0.07	0.03	1.41	1.35	1.98	0.45	0.98	1.09	1.81	0.76
Olsenella	0.63	0.25	0.00	0.03	0.23	0.00	0.08	0.08	0.00	0.00	0.00	0.01	0.70	0.02
Ornithobacterium	0.09	0.00	0.02	0.05	0.01	0.11	0.01	0.04	0.07	0.06	0.03	0.04	0.08	0.04
Oscillibacter	0.75	0.00	0.16	0.49	0.15	0.48	0.19	0.42	0.71	0.41	0.54	0.64	0.78	0.26
Parabacteroides	0.47	0.00	0.46	2.33	0.07	0.10	1.35	1.55	2.19	2.89	3.04	3.76	2.47	1.67
Pauljensenia	0.50	0.21	0.00	0.09	0.31	0.00	0.01	0.07	0.00	0.00	0.04	0.02	0.05	0.11
Phocaeicola	13.18	0.23	8.30	12.16	0.43	15.19	11.87	11.82	15.14	18.98	10.51	8.77	8.98	7.43
Phyllobacterium	0.04	0.23	0.00	0.15	0.39	1.79	0.65	0.25	0.01	0.00	0.04	0.08	0.04	0.09
Porphyromonas	0.00	0.00	0.00	0.00	0.00	0.01	0.00	0.01	0.80	0.00	0.02	0.00	0.00	0.00
Prevotella	0.12	0.30	0.60	0.18	0.13	0.39	0.06	0.21	0.19	0.01	0.08	0.07	0.10	0.01

GENUS	FE-HC	FE-UC-3M	FE-UC-6M	FE-UC-DE	FE-CD-3M	FE-CD-6M	FE-CD-DE	OR-HC	OR-UC-3M	OR-UC-6M	OR-UC-DE	OR-CD-3M	OR-CD-3M	OR-CD-6M
Proteus	0.00	0.00	0.00	0.00	0.00	0.00	0.00	0.00	0.00	0.00	0.00	0.12	0.00	0.00
Pseudopropionibacterium	0.04	0.42	0.00	0.27	0.10	0.00	0.06	0.79	0.00	0.00	0.12	0.13	0.08	0.07
Rhizobium	0.00	0.02	0.00	0.01	0.03	0.19	0.06	0.02	0.00	0.00	0.00	0.01	0.00	0.01
Romboutsia	0.00	0.00	0.29	0.00	0.00	0.00	0.00	0.03	0.02	0.02	0.01	0.01	0.03	0.00
Roseburia	0.67	0.00	0.09	0.18	0.18	0.04	0.34	1.61	1.05	5.52	1.11	2.01	1.60	0.63
Rothia	1.82	3.16	8.85	3.59	3.61	5.09	1.42	3.04	0.01	0.08	0.67	1.97	0.76	4.49
Ruminococcus	0.49	0.00	1.43	0.42	0.06	0.07	0.06	0.59	0.40	1.06	0.63	2.34	1.46	0.22
Schaalia	0.07	0.06	0.00	0.02	0.09	0.00	0.04	0.03	0.00	0.00	0.02	0.01	0.03	0.08
Sinorhizobium	0.00	0.01	0.00	0.01	0.02	0.08	0.03	0.01	0.00	0.00	0.00	0.00	0.00	0.00
Streptococcus	29.25	81.30	41.68	18.69	78.68	31.08	41.04	29.24	0.17	3.33	23.64	18.07	12.57	43.54
Sutterella	0.03	0.00	0.00	0.01	0.01	0.03	0.04	0.02	0.01	0.00	0.01	0.01	0.03	0.00
Veillonella	2.43	1.37	0.63	0.72	1.95	1.23	1.14	0.44	0.06	2.57	0.49	1.04	0.14	0.46

Supplementary Table 3. Result of the binomial test applied to percentages of bacterial families detected in the different groups.

Family	FE-HC	FE-UC-3M	FE-UC-6M	FE-UC-DE	FE-CD-3M	FE-CD-6M	FE-CD-DE	OR-HC	OR-UC-3M	OR-UC-6M	OR-UC-DE	OR-CD-3M	OR-CD-3M	OR-CD-6M
Acidaminococcaceae	0.00	0.00	0.00	0.00	0.00	0.00	0.35	0.00	0.00	0.00	0.00	0.01	0.00	0.00
Actinomycetaceae	12.78	5.26	0.64	2.54	5.16	0.89	4.51	2.40	0.00	0.01	1.14	0.97	1.75	5.19
Aerococcaceae	0.00	0.01	0.00	0.01	0.02	0.00	0.03	0.01	0.00	0.00	0.01	0.00	0.01	0.01
Akkermansiaceae	0.42	0.00	0.00	1.73	0.88	0.00	0.61	0.00	0.53	0.01	0.02	0.79	1.46	0.37
Atopobiaceae	0.66	0.27	0.00	0.03	0.29	0.00	0.09	0.10	0.00	0.00	0.01	0.02	0.85	0.05
Bacteroidaceae	28.61	0.47	26.30	50.24	3.52	39.91	34.84	34.16	57.53	46.00	38.32	35.16	39.90	25.09
Barnesiellaceae	0.10	0.00	0.03	0.07	0.02	0.02	0.03	0.11	0.16	0.07	0.07	0.12	0.16	0.10
Bifidobacteriaceae	2.97	0.00	0.01	5.24	0.04	0.51	0.07	4.42	1.61	4.61	1.88	1.54	1.23	0.34
Brucellaceae	0.00	0.03	0.00	0.02	0.05	0.21	0.07	0.03	0.00	0.00	0.00	0.01	0.00	0.01
Campylobacteraceae	0.01	0.04	0.08	0.01	0.01	0.05	0.00	0.00	0.00	0.27	0.00	0.00	0.01	0.00
Clostridiaceae	0.07	0.00	0.01	0.02	0.01	0.02	0.02	0.02	0.04	0.25	0.03	0.03	0.05	0.03
Coriobacteriaceae	0.05	0.01	0.00	0.05	0.10	0.00	0.05	0.09	0.04	0.03	0.04	0.05	0.05	0.10
Coriobacteriaceae;	0.10	0.00	0.00	0.06	0.09	0.00	0.06	0.24	0.37	0.28	0.26	0.25	0.32	0.37
Desulfovibrionaceae	0.00	0.00	0.00	0.00	0.00	0.00	0.09	0.00	0.05	0.01	0.00	0.06	0.14	0.04
Eggerthellaceae	0.02	0.01	0.06	0.04	0.00	0.01	0.00	0.04	0.07	0.02	0.02	0.10	0.04	0.03
Enterobacteriaceae	0.15	0.12	0.01	0.90	0.55	0.19	2.07	1.51	0.52	2.52	1.65	2.78	0.46	4.17
Enterococcaceae	0.01	0.05	0.01	0.02	0.04	0.01	0.05	0.09	0.01	0.03	0.03	0.02	0.04	0.03

Family	FE-HC	FE-UC-3M	FE-UC-6M	FE-UC-DE	FE-CD-3M	FE-CD-6M	FE-CD-DE	OR-HC	OR-UC-3M	OR-UC-6M	OR-UC-DE	OR-CD-3M	OR-CD-3M	OR-CD-6M
Erysipelotrichaceae	0.34	0.00	0.12	0.10	0.06	0.06	0.07	0.20	0.35	0.69	0.32	0.38	0.42	0.14
Eubacteriaceae	0.17	0.00	0.01	0.07	0.02	0.01	0.04	0.06	0.14	0.14	0.05	0.14	0.07	0.06
Flavobacteriaceae	0.00	0.15	0.16	0.07	0.00	0.12	0.01	0.01	0.00	0.00	0.06	0.00	0.00	0.00
Fusobacteriaceae	0.03	0.25	0.67	0.10	0.11	0.50	0.03	0.01	0.00	0.10	0.17	0.03	0.03	0.03
Hafniaceae	0.00	0.00	0.00	0.00	0.00	0.00	0.00	0.24	0.00	0.00	0.00	0.00	0.00	0.00
Incertaesedis	0.70	2.37	1.16	1.08	1.20	0.30	0.84	2.48	1.07	1.76	2.05	1.59	3.63	1.59
Lachnospiraceae	10.50	0.03	1.90	2.66	1.07	2.29	1.97	5.08	8.94	11.98	8.20	7.87	7.65	2.82
Lachnospiraceae;	0.39	0.00	0.22	0.16	0.12	0.02	0.66	0.17	0.40	0.34	0.17	0.30	0.31	0.09
Methanobacteriaceae	0.00	0.00	0.00	0.00	0.00	0.00	0.00	0.00	0.00	0.00	0.00	0.01	0.00	0.07
Micrococcaceae	1.52	3.06	8.62	3.39	3.47	4.76	1.35	2.83	0.01	0.08	0.61	1.84	0.68	4.14
Morganellaceae	0.00	0.00	0.00	0.00	0.00	0.00	0.00	0.00	0.00	0.00	0.00	0.11	0.00	0.00
Neisseriaceae	0.44	2.14	5.10	2.32	0.30	6.28	0.11	0.13	0.00	0.01	0.48	0.00	0.06	0.19
Odoribacteraceae	0.28	0.01	0.50	0.22	0.07	0.03	1.31	1.24	1.77	0.41	0.89	0.98	1.61	0.69
Oscillospiraceae	5.87	0.01	5.95	3.92	1.18	1.77	3.99	7.67	17.28	14.36	9.99	12.80	16.79	4.74
Oscillospiraceae;	3.46	0.00	0.02	0.01	0.01	0.00	0.05	0.09	0.43	0.08	0.47	0.16	1.58	2.37
Pasteurellaceae	0.29	1.92	4.42	0.97	0.47	4.74	0.70	0.33	0.08	4.94	2.02	0.16	0.28	0.08
Peptostreptococcaceae	0.10	0.01	0.33	0.06	0.04	0.07	0.07	0.09	0.14	0.13	0.09	0.13	0.18	0.07
Phyllobacteriaceae	0.08	0.55	0.00	0.36	0.93	4.21	1.52	0.57	0.02	0.00	0.09	0.18	0.10	0.20
Porphyromonadaceae	0.00	0.00	0.00	0.00	0.00	0.01	0.00	0.01	0.72	0.00	0.02	0.00	0.00	0.00

Family	FE-HC	FE-UC-3M	FE-UC-6M	FE-UC-DE	FE-CD-3M	FE-CD-6M	FE-CD-DE	OR-HC	OR-UC-3M	OR-UC-6M	OR-UC-DE	OR-CD-3M	OR-CD-3M	OR-CD-6M
Prevotellaceae	0.24	0.30	0.61	0.24	0.28	0.40	0.17	0.56	0.35	0.01	0.12	0.21	0.14	0.03
Propionibacteriaceae	0.27	1.31	0.00	1.03	0.62	0.19	1.05	2.58	0.00	0.00	0.41	0.43	0.45	0.35
Rhizobiaceae	0.02	0.13	0.00	0.09	0.22	1.20	0.38	0.14	0.01	0.00	0.02	0.05	0.02	0.05
Rikenellaceae	1.82	0.00	0.64	1.26	0.70	0.14	0.60	2.57	5.02	2.78	4.68	9.41	5.39	4.23
Selenomonadaceae	0.00	0.00	0.00	0.00	0.06	0.00	0.86	0.02	0.00	0.00	0.00	0.00	0.01	0.00
Streptococcaceae	24.71	80.12	41.29	17.86	76.07	29.54	38.79	27.71	0.16	3.02	22.28	16.89	11.62	40.10
Sutterellaceae	0.07	0.00	0.00	0.03	0.03	0.09	0.09	0.04	0.02	0.00	0.02	0.02	0.07	0.01
Tannerellaceae	0.61	0.00	0.45	2.27	0.10	0.12	1.30	1.45	2.00	2.61	2.81	3.43	2.23	1.55
Veillonellaceae	2.07	1.36	0.66	0.71	2.08	1.23	1.09	0.43	0.07	2.40	0.46	0.98	0.13	0.44
Weeksellaceae	0.07	0.00	0.02	0.05	0.01	0.11	0.01	0.04	0.06	0.06	0.03	0.04	0.07	0.04

Supplementary Table 4. Biodiversity estimators (richness and diversity) by experimental group

TAXA	*Group*	*Group number*	*JACKNIFE2*	*CHAO*	*S*	*H*	*simp*	*invsimp*	*alpha*	*Eveness: J Pielou's*	***Hill test*** *Group number, type, q p-value*
family	*FE*	*gr1*	*48,92*	*45 1,897234*	*45,00*	*1,90*	*0,73*	*3,66*	*2,53*	*0,50*	
family	*OR*	*gr2*	*45,04*	*46 2,120656*	*46,00*	*2,12*	*0,79*	*4,86*	*2,48*	*0,55*	*gr2 - gr1 0 0.0000*<br>*gr2 - gr1 1 0.0132*<br>*gr2 - gr1 2 0.0906*
genus	*FE*	*gr1*	*76,92*	*73,99*	*73,00*	*2,02*	*0,74*	*3,78*	*4,24*	*0,47*	
genus	*OR*	*gr2*	*73,04*	*74,00*	*74,00*	*2,41*	*0,84*	*6,23*	*4,13*	*0,56*	*gr2 - gr1 0 0.0000*<br>*gr2 - gr1 1 0.0000*<br>*gr2 - gr1 2 0.0004*
specie	*FE*	*gr1*	*139,92*	*136,99*	*136,00*	*3,49*	*0,94*	*17,01*	*8,32*	*0,71*	
specie	*OR*	*gr2*	*137,04*	*138,00*	*138,00*	*3,62*	*0,95*	*20,59*	*8,04*	*0,74*	*gr2 - gr1 0 0.0000*<br>*gr2 - gr1 1 0.6166*<br>*gr2 - gr1 2 0.9868*
family	*FE-HC*	*gr1*	*113,36*	*125,08*	*125,00*	*3,60*	*0,95*	*20,47*	*8,80*	*0,75*	
family	*FE-UC-3M*	*gr2*	*145,65*	*134,60*	*126,00*	*2,64*	*0,87*	*7,45*	*9,35*	*0,55*	
family	*FE-UC-6M*	*gr3*	*133,79*	*121,46*	*105,00*	*3,27*	*0,93*	*14,47*	*8,79*	*0,70*	
family	*FE-UC-DE*	*gr4*	*128,12*	*134,88*	*134,00*	*3,40*	*0,93*	*14,90*	*9,09*	*0,69*	
family	*FE-CD-3M*	*gr5*	*141,17*	*136,71*	*133,00*	*2,65*	*0,85*	*6,49*	*9,05*	*0,54*	
family	*FE-CD-6M*	*gr6*	*143,48*	*130,94*	*118,00*	*3,04*	*0,91*	*10,86*	*9,39*	*0,64*	

TAXA	Group	Group number	JACKNIFE2	CHAO	S	H	simp	invsimp	alpha	Eveness: J Pielou's	Hill test Group number, type, q p-value
family	FE-CD-DE	gr7	135,53	133,69	130,00	3,27	0,93	14,80	8,52	0,67	
family	OR-HC	gr8	141,25	138,85	137,00	3,52	0,95	18,24	9,31	0,72	
family	OR-UC-3M	gr9	134,46	134,45	133,00	2,89	0,91	10,80	8,88	0,59	
family	OR-UC-6M	gr10	112,98	112,43	112,00	3,13	0,92	12,71	7,43	0,66	
family	OR-UC-DE	gr11	129,91	136,57	136,00	3,54	0,95	21,75	8,84	0,72	
family	OR-CD-3M	gr12	139,98	138,38	137,00	3,54	0,95	21,23	8,92	0,72	
family	OR-CD-6M	gr13	135,57	137,31	136,00	3,52	0,95	18,23	9,16	0,72	
family	OR-CD-DE	gr14	141,20	139,48	138,00	3,41	0,94	16,05	9,00	0,69	
genus	FE-HC	gr1	61,22	65,06	65,00	2,35	0,84	6,21	4,33	0,56	
genus	FE-UC-3M	gr2	74,75	70,56	67,00	0,92	0,33	1,50	4,63	0,22	
genus	FE-UC-6M	gr3	54,26	51,20	49,00	2,00	0,77	4,35	3,79	0,51	
genus	FE-UC-DE	gr4	65,00	71,40	71,00	2,16	0,79	4,70	4,56	0,51	
genus	FE-CD-3M	gr5	79,98	77,49	70,00	1,11	0,38	1,60	4,48	0,26	
genus	FE-CD-6M	gr6	71,16	65,58	61,00	2,05	0,80	5,04	4,52	0,50	
genus	FE-CD-DE	gr7	74,53	74,15	70,00	1,99	0,76	4,09	4,35	0,47	
genus	OR-HC	gr8	79,97	78,56	73,00	2,34	0,83	5,88	4,70	0,55	
genus	OR-UC-3M	gr9	67,82	70,55	70,00	1,85	0,72	3,55	4,46	0,44	gr9 - gr1 0 0.0066<br>gr9 - gr2 0 0.0080<br>gr9 - gr 3 0 0.0044<br>gr 9 - gr 6 0 0.0000

TAXA	***Group***	***Group number***	***JACKNIFE2***	***CHAO***	***S***	***H***	***simp***	***invsimp***	***alpha***	***Eveness:***<br>***J Pielou's***	***Hill test***<br>*Group number, type, q*<br>*p-value*
genus	*OR-UC-6M*	*gr10*	*55,57*	*54,00*	*54,00*	*2,29*	*0,83*	*6,01*	*3,40*	*0,57*	*gr 10 - gr 3 0 0.0608*<br>*gr 10 - gr 6 0 0.0070*
genus	*OR-UC-DE*	*gr11*	*67,86*	*72,48*	*72,00*	*2,31*	*0,83*	*5,78*	*4,46*	*0,54*	*gr11 - gr1 0 0.0208*<br>*gr11 - gr2 0 0.0256*<br>*gr 11 - gr 3 0 0.0150*<br>*gr 11 - gr 6 0 0.0010*
genus	*OR-CD-3M*	*gr12*	*77,49*	*77,13*	*73,00*	*2,47*	*0,85*	*6,90*	*4,51*	*0,58*	*gr12 - gr1 0 0.0040*<br>*gr12 - gr2 0 0.0052*<br>*gr 12 - gr 3 0 0.0032*<br>*gr 12 - gr 6 0 0.0000*
genus	*OR-CD-6M*	*gr13*	*71,06*	*72,87*	*72,00*	*2,41*	*0,83*	*5,90*	*4,61*	*0,56*	*gr13 - gr1 0 0.0072*<br>*gr13 - gr2 0 0.0102*<br>*gr 13 - gr 3 0 0.0060*<br>*gr 13 - gr 6 0 0.0000*
genus	*OR-CD-DE*	*gr14*	*76,98*	*75,38*	*74,00*	*2,05*	*0,76*	*4,15*	*4,57*	*0,48*	*gr 14 - gr 3 0 0.0482*<br>*gr 14 - gr 6 0 0.0028*
Species	*FE-HC*	*gr1*	*113,36* *125,08*	*125,00*	*3,60*		*0,95*	*20,47*	*8,80*	*0,75*	
Species	*FE-UC-3M*	*gr2*	*145,65* *134,60*	*126,00*	*2,64*		*0,87*	*7,45*	*9,35*	*0,55*	
Species	*FE-UC-6M*	*gr3*	*133,79* *121,46*	*105,00*	*3,27*		*0,93*	*14,47*	*8,79*	*0,70*	
Species	*FE-UC-DE*	*gr4*	*128,12* *134,88*	*134,00*	*3,40*		*0,93*	*14,90*	*9,09*	*0,69*	

TAXA	Group	Group number	JACKNIFE2		CHAO	S	H	simp	invsimp	alpha	Eveness:<br>J Pielou's	Hill test<br>Group number, type, q<br>p-value
Species	*FE-CD-3M*	*gr5*	*141,17*	*136,71*	*133,00*	*2,65*		*0,85*	*6,49*	*9,05*	*0,54*	
Species	*FE-CD-6M*	*gr6*	*143,48*	*130,94*	*118,00*	*3,04*		*0,91*	*10,86*	*9,39*	*0,64*	
Species	*FE-CD-DE*	*gr7*	*135,53*	*133,69*	*130,00*	*3,27*		*0,93*	*14,80*	*8,52*	*0,67*	
Species	*OR-HC*	*gr8*	*141,25*	*138,85*	*137,00*	*3,52*		*0,95*	*18,24*	*9,31*	*0,72*	
Species	*OR-UC-3M*	*gr9*	*134,46*	*134,45*	*133,00*	*2,89*		*0,91*	*10,80*	*8,88*	*0,59*	*gr 9 - gr 1 0 0.0000*<br>*gr 9 - gr 2 0 0.0002*<br>*gr 9 - gr 3 0 0.0006*<br>*gr 9 - gr 5 0 0.0020*<br>*gr 9 - gr 6 0 0.0000*<br>*gr 9 - gr 7 0 0.0076*
Species	*OR-UC-6M*	*gr10*	*112,98*	*112,43*	*112,00*	*3,13*		*0,92*	*12,71*	*7,43*	*0,66*	*gr 10 - gr 1 0 0.0064*<br>*gr 10 - gr 2 0 0.0074*<br>*gr 10 - gr 3 0 0.0188*<br>*gr 10 - gr 5 0 0.0396*<br>*gr 10 - gr 6 0 0.0002*<br>*gr 10 - gr 7 0 0.0996*
species	*OR-UC-DE*	*gr11*	*129,91*	*136,57*	*136,00*	*3,54*		*0,95*	*21,75*	*8,84*	*0,72*	*gr 11 - gr 1 0 0.0030*<br>*gr 11 - gr 2 0 0.0040*<br>*gr 11 - gr 3 0 0.0124*<br>*gr 11 - gr 5 0 0.0224*<br>*gr 11 - gr 6 0 0.0000*<br>*gr 11 - gr 7 0 0.0628*

TAXA	*Group*	*Group number*	*JACKNIFE2*		*CHAO*	*S*	*H*	*simp*	*invsimp*	*alpha*	*Eveness: J Pielou's*	*Hill test*<br>*Group number, type, q p-value*
Species	*OR-CD-3M*	*gr12*	*139,98*	*138,38*	*137,00*		*3,54*	*0,95*	*21,23*	*8,92*	*0,72*	*gr 12 - gr 1 0 0.0020*<br>*gr 12 - gr 2 0 0.0026*<br>*gr 12 - gr 3 0 0.0098*<br>*gr 12 - gr 5 0 0.0188*<br>*gr 12 - gr 6 0 0.0000*<br>*gr 12 - gr 7 0 0.0508*
Species	*OR-CD-6M*	*gr13*	*135,57*	*137,31*	*136,00*		*3,52*	*0,95*	*18,23*	*9,16*	*0,72*	*gr 13 - gr 1 0 0.0002*<br>*gr 13 - gr 2 0 0.0004*<br>*gr 13 - gr 3 0 0.0030*<br>*gr 13 - gr 5 0 0.0052*<br>*gr 13 - gr 6 0 0.0000*<br>*gr 13 - gr 7 0 0.0196*
Species	*OR-CD-DE*	*gr14*	*141,20*	*139,48*	*138,00*		*3,41*	*0,94*	*16,05*	*9,00*	*0,69*	*gr 14 - gr 6 0 0.0562*

The controls for the different groups of samples are shown in green.

Supplementary Table 5. Summary of the statistical analysis of the bacterial consortia in the different groups during the clinical evolution of the IBD patients using complex networks.

Group	Taxonomy	Complexity Index (CI)	CI(EDGES>1)	Number of connections within network (nc), Network diameter / effective size of the network (nd), Number of formed groups (g), Connection quality (cq)
HC-FE	Species	5.80	6.80	41nc, 5nd, 2g, connect (2g all-all) cq
	Families	5.80	6.80	22nc, NAnd, 2g, connect (2g all-all) cq
	Genera	3.90	5.30	11nc, 1g, connect (2g all-all) cq
FE-CD-DE	Species	4.40	6.40	14 nc, nd= 4, 2g, connected cq
	Families	2.10	2.60	15 nc, NAnd, 3g, med connected cq
	Genera	2.20	3.40	5 nc, 1g, few connected cq
FE-CD-3M	Species	3.90	4.30	51 nc, nd=5, 5g, connected (1g all-all) cq
	Families	1.50	4.00	30 nc, NAnd, 2g, med-connected (1g all-all) cq
	Genera	1.60	2.90	16 nc, 1g (1g all-all) cq
FE-CD-6M	Species	2.50	3.10	74 (rm=2) nc, nd=2, 1g, connected (1g all-all) cq
	Families	1.80	2.60	45 nc, NAnd , 1g, connected (1g all-all) cq
	Genera	2.06	3.00	29 nc, NAnd , 1g, connected (1g all-all) cq
FE-UC-DE	Species	2.90	4.30	38 nc, nd=6, 4g, connected (1 grupo all-all) cq
	Families	1.60	2.00	22 nc, NAnd , 3g, connected (1g all-all) cq

Group	Taxonomy	Complexity Index (CI)	CI(EDGES>1)	Number of connections within network (nc), Network diameter / effective size of the network (nd), Number of formed groups (g), Connection quality (cq)
	Genera	1.80	2.60	10 nc, 1-2g, connected cq
FE-UC-3M	Species	2.20	2.90	42 nc, nd=2, 1g, med-connected (1g all-all) cq
	Families	1.70	2.30	22 nc, NAnd, 1g, connected (1g all-all) cq
	Genera	2.50	3.30	16 nc, NAnd , 1g, connected (1g all-all) cq
FE-UC-6M	Species	1.80	2.40	55 nc, NAnd , 1g, connected (1g all-all) cq
	Families	1.30	2.00	40 nc, NAnd , 1g, connected (1g all-all) cq
	Genera	1.30	2.20	20 nc, NAnd , 1g, connected (1g all-all) cq
HC-OR	Species	1.84	3.00	25 nc, nd=4, 2-3g, connect cq
	Families	1.90	2.40	10 nc, NAnd, 2g, few connections cq
	Genera	1.90	2.50	4 nc, 1g, all-all cq
OR-CD-DE	Species	1.67	2.70	12 nc, NAnd , 1g, all-all cq
	Families	1.30	2.00	6 nc, NAnd , 1g, all-all cq
	Genera	1.40	2.00	4 nc, NAnd, 1g, all-all cq
OR -CD-3M	Species	1.20	3.00	19 nc, NAnd, 2g, connected (1g all-all) cq
	Families	1.10	2.00	5 nc, NAnd, 1g, connected (1g all-all) cq

Group	Taxonomy	Complexity Index (CI)	CI(EDGES>1)	Number of connections within network (nc), Network diameter / effective size of the network (nd), Number of formed groups (g), Connection quality (cq)
	Genera	1.20	2.20	5 nc, NAnd, 1g, connected (1g all-all) cq
OR -CD-6M	Species	1.50	2.00	32 nc, NAnd, 1g, connected (1g all-all) cq
	Families	1.20	2.50	12 nc, NAnd, 1g, connected (1g all-all) cq
	Genera	1.30	2.20	5 nc, NAnd, 1g, connected, 1g all-all cq
OR -UC-DE	Species	1.75	2.36	35 nc, NAnd, 3g, connected (1g all-all) cq
	Families	1.25	2.50	17 nc, NAnd, 2g, connected (2g all-all) cq
	Genera	1.30	2.30	5 nc, NAnd, 1g, connected (1g all-all) cq
OR -UC-3M	Species	1.00	2.00	12 nc, NAnd, 2g, med-connected (1g all-all) cq
	Families	1.00	2.00	6 nc, NAnd, 1g, med-connected (0g all-all) cq
	Genera	1.30	2.40	5 nc, NAnd, 1g, connected (1g all-all) cq
OR -UC-6M	Species	1.10	2.00	30 nc, NAnd, 2g, connected (1g all-all) cq
	Families	1.10	2.00	14 nc, NAnd, 1g, connected (1g all-all) cq
	Genera	1.00	2.00	9 nc, NAnd, 1g, connected, 1g all-all cq

2. References

[1] Frías-López J, Durán-Pinedo A. Effect of periodontal pathogens on the metatranscriptome of a healthy multispecies biofilm model. J Bacteriol. (2012) 194 (8):2082–95. doi: 10.1128/JB.06328-11

[2] Rodríguez-Casado CI, Monleón-Getino T. A new R library for discriminating groups based on abundance profile and biodiversity in microbiome metagenomic matrices. Int J Sci Eng Res. (2016) 7(10):243–53.

[3] Pascal V, Pozuelo M, Borruel N, Casellas F, Campos D, Santiago A, et al. A microbial signature for Crohn's disease. Gut. (2017) 66(5):813–22. doi: 10.1136/ gutjnl-2016-313235

[4] Seyed Tabib NS, Madgwick M, Sudhakar P, Verstockt B, Korcsmaros T, Vermeire. S. Big data in IBD: big progress for clinical practice. Gut. (2020) 69(8):1520–32. doi: 10.1136/gutjnl-2019-320065

[5] Erickson AR, Cantarel BL, Lamendella R, Darzi Y, Mongodin EF, Pan C, et al. Integrated metagenomics/metaproteomics reveals human host-microbiota signatures of Crohn's disease. Plos One. (2012) 7(11):1–14. doi: 10.1371/journal.pone.0049138

[6] Fraser-Liggett CM. Baltimore metagenomic analysis of the structure and function of the human gut microbiota in Crohn's disease. Natureprecedings. (2010): 1-5. doi: 10.1038/npre.2010.4958.1

[7] Fouhy F, Ross RP, Fitzgerald GF, Stanton C, Cotter PD. Composition of the early intestinal microbiota: knowledge, knowledge gaps and the use of high-throughput sequencing to address these gaps. Gut Microbes. 2012 May-Jun;3(3):203-20. doi: 10.4161/gmic.20169. Epub 2012 May 1. PMID: 22572829; PMCID: PMC3427213

[8] Mueller NT, Bakacs E, Combellick J, Grigoryan Z, Dominguez-Bello MG. The infant microbiome development: mom matters. Trends Mol Med. 2015 Feb;21(2):109-17. doi: 10.1016/j.molmed.2014.12.002. Epub 2014 Dec 11. PMID: 25578246; PMCID: PMC4464665.

[9] Fitzgerald, R. S., Sanderson, I. R. and Claesson, Marcus J. (2021) 'Paediatric inflammatory bowel disease and its relationship with the microbiome', Microbial Ecology, 82, pp. 833–844. doi: 10.1007/s00248- 021-01697-9

[10] Docktor MJ, Paster BJ, Abramowicz S, Ingram J, Wang YE, Correll M, Jiang H, Cotton SL, Kokaras AS, Bousvaros A. Alterations in diversity of the oral microbiome in pediatric inflammatory bowel disease. Inflamm Bowel Dis. 2012 May;18(5):935-42. doi: 10.1002/ibd.21874. Epub 2011 Oct 10. PMID: 21987382; PMCID: PMC4208308.

[11] World Register of marine Species. Measurements of biodiversity. Word Register of Introduced marine Species. (2022). Available at: https://www.marinespecies.org/ introduced/wiki/Measurements_of_biodiversity

[12] Monleón-Getino T. Library for R BDSBIOST3: Machine Learning and Advanced Statistical Methods for Omic categorical analysis and others [WWW Document]. (2020). Available at: https://github.com/amonleong/BDSbiost3

[13] Pallmann P, Schaarschmidt F, Hothorn LA, Fischer C, Nacke H, Priesnitz KU, Schork NJ. Assessing group differences in biodiversity by simultaneously testing a user-defined selection of diversity indices. Mol Ecol Resour. (2012) 12(6):1068–78. doi: 10.1111/1755-0998.12004

[14] R Core Team (2021). R: A language and environment for statistical computing. R Foundation for Statistical Computing, Vienna, Austria. URL https://www.R-project.org/.

[15] Monleón-Getino, A., Rodríguez-Casado, C., Mendez-Viera, J., 2017. How to calculate number of samples in the design of pre/pro-biotics studies (metagenomic studies). In: How to Calculate Number of Samples in the Design of Pre/pro-Biotics Studies. Seville. III WorkShop Insa-UB (2017, Barcelona, Spain). https://diposit.ub.edu/dspace/bitstream/2445/120104/1/Monleon_Getino_2.pdf : 1-2. https://doi.org/10.13140/RG.2.2.27611.67367.

[16] Monleón-Getino T, Frías-Lopez J. A priori estimation of sequencing effort in complex microbial metatranscriptomes. Ecol Evol. (2020) 10(23):13382–94. doi: 10. 1002/ece3.6941

[17] Pascual-Benito M, Balleste E, Monleón-Getino T, Urmeneta J, Blanch AR, García-Aljaro C, et al. Impact of treated sewage effluent on the bacterial community composition in an intermittent Mediterranean stream. Environ Pollut. (2020) 266:115254. doi: 10.1016/j.envpol.2020.115254

[18] Yang Z, Algesheimer R, Tessone C. A comparative analysis of community detection algorithms on artificial networks. Sci Rep. (2016) 6:30750. doi: 10.1038/ srep30750

[19] Díaz-Garrido N, Lozano CP, Kreth J, Giacaman RA. Competition and caries on enamel of a dual-species biofilm model with Streptococcus mutans and Streptococcus sanguinis. Appl Environ Microbiol. (2020) 86(21):e01262–20. doi: 10.1128/AEM. 01262-20

[20] Treerat P, Redanz U, Redanz S, Giacaman RA, Merritt J, Kreth J. Synergism between Corynebacterium and Streptococcus sanguinis reveals new interactions between oral commensals. ISME J. (2020) 14(5):1154–69. doi: 10.1038/s41396-020- 0598-2

[21] Brito F, Zaltman C, Carvalho AT, Fischer RG, Persson R, Gustafsson A, et al. Subgingival microflora in inflammatory bowel disease patients with untreated periodontitis. Eur J Gastroenterol Hepatol. (2013) 25(2):239–45. doi: 10.1097/MEG. 0b013e32835a2b70 [21]

[22] Cao Y, Wang Z, Yan Y, Ji L, He J, Xuan B, et al. Enterotoxigenic bacteroidesfragilis promotes intestinal inflammation and malignancy by inhibiting exosome-packaged miR-149-3p. Gastroenterology. (2021) 161(5):1552–1566.e12. doi: 10.1053/j.gastro.2021.08.003

[23] Zamani S, Hesam Shariati S, Zali MR, Asadzadeh Aghdaei H, Sarabi Asiabar A, Bokaie S, et al. Detection of enterotoxigenic Bacteroides fragilis in patients with ulcerative colitis. Gut Pathog. (2017) 9:53. doi: 10.1186/s13099-017-0202-0

[24] Chu H, Khosravi A, Kusumawardhani IP, Kwon AH, Vasconcelos AC, Cunha LD, et al. Gene-microbiota interactions contribute to the pathogenesis of inflammatory

bowel disease. Science. (2016) 352(6289):1116–20. doi: 10.1126/ science.aad 9948

[25] Li S, Wang C, Zhang C, Luo Y, Cheng Q, Yu L, et al. Evaluation of the effects of different Bacteroides vulgatus strains against DSS-induced colitis. J Immunol Res. (2021) 2021:9117805. doi: 10.1155/2021/9117805

[26] Wang C, Xiao Y, Yu L, Tian F, Zhao J, Zhang H, et al. Protective effects of different Bacteroides vulgatus strains against lipopolysaccharide-induced acute intestinal injury, and their underlying functional genes. J Adv Res. (2021) 36:27–37. doi: 10.1016/j.jare.2021.06.012

[27] Bloom SM, Bijanki VN, Nava GM, Sun L, Malvin NP, Donermeyer DL, et al. Commensal Bacteroides species induce colitis in host-genotype-specific fashion in a mouse model of inflammatory bowel disease. Cell Host Microbe. (2011) 9 (5):390–403. doi: 10.1016/j.chom.2011.04.009
